# Successful year-round mainstream partial nitritation anammox: Assessment of effluent quality, performance and N_2_O emissions

**DOI:** 10.1016/j.wroa.2022.100145

**Published:** 2022-06-16

**Authors:** D. Hausherr, R. Niederdorfer, H. Bürgmann, M.F. Lehmann, P. Magyar, J. Mohn, E. Morgenroth, A. Joss

**Affiliations:** a Eawag, Swiss Federal Institute of Aquatic Science and Technology, Dübendorf 8600, Switzerland; b Eawag, Swiss Federal Institute of Aquatic Science and Technology, Kastanienbaum, 6047, Switzerland; c Department of Environmental Sciences, University of Basel, Aquatic and Isotope Biogeochemistry, Basel 4056, Switzerland; d Empa, Swiss Federal Institute for Materials Science and Technology, Laboratory for Air Pollution / Environmental Technology, Dübendorf 8600, Switzerland; e ETH Zürich, Institute of Environmental Engineering, Zürich 8093, Switzerland

**Keywords:** Two-stage, Pilot-scale, PNA, Isotopes, Municipal wastewater

## Abstract

For two decades now, partial nitritation anammox (PNA) systems were suggested to more efficiently remove nitrogen (N) from mainstream municipal wastewater. Yet to date, only a few pilot-scale systems and even fewer full-scale implementations of this technology have been described. Process instability continues to restrict the broad application of PNA. Especially problematic are insufficient anammox biomass retention, the growth of undesired aerobic nitrite-oxidizers, and nitrous oxide (N_2_O) emissions. In this study, a two-stage mainstream pilot-scale PNA system, consisting of three reactors (carbon pre-treatment, nitritation, anammox - 8 m^3^ each), was operated over a year, treating municipal wastewater. The aim was to test whether both, robust autotrophic N removal and high effluent quality, can be achieved throughout the year. A second aim was to better understand rate limiting processes, potentially affecting the overall performance of PNA systems. In this pilot study, excellent effluent quality, in terms of inorganic nitrogen, was accomplished (average effluent concentrations: 0.4 mgNH_4_-N/L, 0.1 mgNO_2_-N/L, 0.9 mgNO_3_-N/L) even at wastewater temperatures previously considered problematic (as low as 8 °C). N removal was limited by nitritation rates (84 ± 43 mgNH_4_-N/L/d), while surplus anammox activity was observed at all times (178 ± 43 mgN/L/d). Throughout the study, nitrite-oxidation was maintained at a low level (<2.5% of ammonium consumption rate). Unfortunately, high N_2_O emissions from the nitritation stage (1.2% of total nitrogen in the influent) were observed, and, based on natural isotope abundance measurements, could be attributed to heterotrophic denitrification. *In situ* batch experiments were conducted to identify the role of dissolved oxygen (DO) and organic substrate availability in N_2_O emission-mitigation. The addition of organic substrate, to promote complete denitrification, was not successful in decreasing N_2_O emission, but increasing the DO from 0.3 to 2.9 mgO_2_/L decreased N_2_O emissions by a factor of 3.4.

## Highlights

• Mainstream PNA achieved excellent effluent quality (TIN < 2 mgN/L).
• Good performance was maintained even at wastewater temperatures < 15 °C.
• An average N_2_O emission factor of 1.2% was measured over a full year.
• Natural isotope abundance measurements indicate het. denitrification as N_2_O source.

## Graphical abstract

**Figure:**
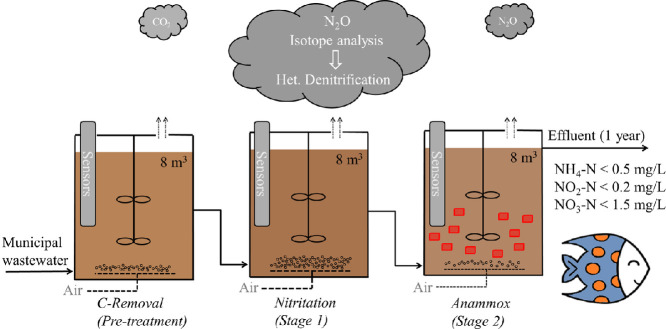

## 1 Introduction

Anammox-based mainstream N removal has multiple advantages over conventional nitrification/denitrification (N/D) systems commonly used in wastewater treatment. Firstly, the anammox metabolism produces only 0.07–0.11 mgNO_3_-N per mg NH_4_-N oxidized, thus less organic substrate is needed for its denitrification to N_2_ (Lotti et al., 2014; Strous et al., 1998). In turn, a greater fraction of organic substrate can be valorized, e.g., used for bio-plastic production or bio-gas generation (Siegrist et al., 2008). In addition, to reach low N effluent concentrations (<8 mgTN/L), no additional organic substrate is required, reducing the operation costs (EPA, 2013). Furthermore, energy consumption for additional aeration can be lowered, if the organic substrate is treated anaerobically (Daigger, 2014; Shin et al., 2021).

The first step in many mainstream PNA systems is to capture most organic matter (carbon depletion), since the organic substrate requirements (for nitrate denitrification) are reduced. Carbon capture is achieved with various approaches, such as high rate activated sludge (HRAS) systems (Rahman et al., 2019; Wett et al., 2013), chemically enhanced precipitation (Taboada-Santos et al., 2020), or micro-sieving (Paulsrud et al., 2014; Rusten et al., 2016).

After carbon depletion, N removal by PNA can be performed in a single combined PNA stage or in a two-stage process (i.e., partial nitritation followed by anammox). In recent years, there has been significant debate regarding the preference of one- versus two-stage systems (one stage: (Han et al., 2018; Li et al., 2016; Pérez et al., 2014; Schoepp et al., 2018; Vlaeminck et al., 2009); two-stage: (Jung et al., 2021; Kouba et al., 2017; Kowalski et al., 2019; Poot et al., 2016)). Single stage PNA results in lower peak nitrite concentrations because nitrite is, continuously or intermittently, reduced by anammox bacteria (AMX). Low nitrite concentrations have two distinct advantages. Firstly, they reduce the growth rate of unwanted nitrite oxidizing bacteria (NOB). Secondly, they help to keep nitrous oxide (N_2_O) production low (Chen et al., 2020; Ma et al., 2017). On the other hand, reaching low effluent ammonium concentrations (<5 mgNH_4_-N/L) can be problematic in single stage systems. At low ammonium concentrations, the growth rate of ammonium oxidizing bacteria (AOB) slows down, which complicates the out-competition of NOB (Corbalá-Robles et al., 2016; Poot et al., 2016). Additionally, two trade-offs need to be addressed in single-stage systems. On the one hand, a long solids retention time (SRT) is required to retain the slow growing AMX while on the other hand NOB need to be washed out. The second trade-off is related to the need for dissolved oxygen (DO) by AOB and the inhibiting effect of DO on AMX (Agrawal et al., 2018). In two-stage PNA systems, NOB suppression and AOB enhancement (stage one) as well as anammox retention (stage two) can be addressed separately.

Over the last 15 years, many publications have reported on working PNA systems in lab-scale reactors and a plethora of strategies to limit NOB growth have been proposed (reviewed in Agrawal et al., 2018; Cao et al., 2017; Li et al., 2018). Yet, only sporadic reports of pilot- or full-scale mainstream anammox installations can be found.

Four pilot- or full-scale mainstream anammox systems reported in the literature (**St**rass WWTP, **M**arlisborg WWTP, **D**okhaven WWTP and **Sj**ölunda WWTP) are briefly discussed here in light of the potential challenges of full-scale PNA (Gustavsson et al., 2020; Hoekstra et al., 2019; Kamp et al., 2019; Weissenbacher et al., 2013; Wett et al., 2013). During the reported study periods, they were operated as single stage PNA systems and three of them (**St, D, Sj**) received pretreated wastewater (carbon removal in an HRAS reactor). Anammox biomass retention was achieved through hydrocyclones (**St**), gravitational settling of granulated biomass (**D**) or biofilm formation on carriers (**Sj**). N removal rates were on the order of 50–600 mgN/L/d. However, in (**St, D, Sj**) high ammonium effluent concentrations were measured (4–10 mgNH_4_-N/L). Furthermore, significant unwanted nitrate production by NOB was observed in some of the plants (**St, D, Sj**), and occasionally, problematic nitrite concentrations of 0.2–1 and 1–6 mgNO_2_-N/L were measured in the effluent of **Sj** and **St**, respectively. In **D** and **Sj**, nitritation instability due to high organic substrate loading was observed. For **M** it was demonstrated that the addition of sidestream anammox granules to a nitrification/denitrification basin contributed only 1% to the observed N removal. Lastly, in **St**, the switch from N/D to deammonification led to an increase in N_2_O emissions.

As mentioned above, the unifying feature of all four WWTPs presented, is that they were operated as single stage PNAs. Refurbishing old WWTPs to single stage PNA systems is simpler and cheaper, since no new basins are needed. Moreover, immediate nitrite consumption by AMX should limit N_2_O emissions, which is critical for the greenhouse gas footprint of WWTPs, and reduces NOB growth rates. However, two common issues prevailed in these single stage PNA trials. (1) High ammonium concentrations in the effluent. Strict ammonium effluent requirements of <2 mgNH_4_-N/L are becoming more common (in Switzerland, ammonium effluent concentrations are not allowed to exceed 2 mgNH_4_-N/L in a 24 h average effluent sample (GeSchV, 2021, Art. 6 Abs. (1)). Achieving low effluent ammonium concentration will thus be a basic requirement for future PNA systems. (2) Unwanted NOB activity. The imminent risk of re-occurring NOB growth, and the consequent demand for operational changes, jeopardizes the long-term stability of the nitritation stage. In addition, as has been documented in **D** and **Sj**, increased organic loading can pose a challenge. For example, organic substrate may be used by denitrifying bacteria, thus consuming nitrite that would otherwise be available for AMX. Moreover, AOB performance can deteriorate in response to increased organic loading, possibly because of inhibiting effects of certain organic compounds, or due to competition for DO with heterotrophs. N_2_O emissions were only quantified in **St**, where they increased from 0.3 to 1% of total nitrogen (TN) in the influent after the switch from N/D to mainstream anammox-based N removal. Given the 298 fold greater greenhouse gas potential of N_2_O with respect to CO_2_, even a small increase in N_2_O emissions can drastically alter the climate impact of wastewater treatment, and thus negate any sustainability gains achieved through reduced aeration or decreased organic substrate dosing (Liao et al., 2020).

In this study, we established a pilot-scale mainstream two-stage PNA system with prior C-removal and monitored its performance over one year. The long-term observational data presented here, allowed a clear attribution of the rate limiting steps, process disruptions and N_2_O emissions to either the nitritation or anammox stage. In addition, N_2_O production pathways were studied using stable isotopes ratio measurements, and strategies to mitigate N_2_O emissions were investigated experimentally. Moreover, this study explored whether limitations observed in previously published pilot-scale mainstream anammox studies (see above) can be overcome in a two-stage PNA system. Can low ammonium effluent concentrations be reached (<1 mgNH_4_-N/L)? Is a higher degree of NOB suppression achievable? Is organic matter that escapes from the pretreatment stage as harmful to two-stage systems, as observed for one-stage systems?

## 2 Material and methods

### 2.1 Pilot-scale wastewater treatment plant

The pilot-scale WWTP is directly fed from the sewer system of the municipality of Dübendorf, Switzerland. Raw wastewater is pumped from the sewer, and mechanically pretreated (5 mm punched-hole sieve followed by oil and sand trap) before it enters a primary settler (PS), which has a hydraulic retention time of 0.5–1 h. Wastewater composition after the PS is provided in Table 1 (taken from Hausherr et al., 2022). From the PS, wastewater is fed to the first reactor (R1-Carb).

**Table 1:** Municipal wastewater characterization after the primary settler (PS) and after pretreatment (R-Carb). Chemical oxygen demand (COD), soluble COD (sCOD), NH_4_^+^, NO_2_^−^, NO_3_^−^, PO_4_^3−^, total nitrogen (TN) and total phosphorus (TP) average values (*n* = 8) and standard deviations are presented. TN and TP were only measured in the effluent of the PS.

Effluent	COD[mg/L]	sCOD[mg/L]	TN[mg/L]	NH_4_-N[mg/L]	NO_2_-N[mg/L]	NO_3_-N[mg/L]	PO_4_-P[mg/L]	TP[mg/L]
PS	469 ± 235	277 ± 189	47 ± 12	25 ± 7	0.3 ± 0.03	0.6 ± 0.5	1.7 ± 1.2	5.2 ± 1.6
R-Carb	137 ± 81	51 ± 22	-	20 ± 8	0.3 ± 0.1	0.4 ± 0.5	1.2 ± 0.5	-

Table 2 presents a summary of the SBR cycles of the three sequential reactors (R1-Carb, R2-PN and R3-AMX). They are all operated as SBRs with linked cycles. Step 8, the idle phase, was used to synchronize the reactors. A volume exchange of ∼90% was carried out for each SBR cycle. To achieve such a high volume exchange, the reactors were bottom-fed (i.e., fresh wastewater was pumped in at the bottom of the reactor and treated wastewater was displaced at the top). A brief illustration of the sequential feeding process is shown in the supporting information (SI, Fig. S1). The SBR cycles were automated and controlled in a supervisory control and data acquisition software (Citect, Australia). All SBR steps with a fixed length were terminated after the specified amount of time had elapsed (Table 2). The control for the variable steps are described for each reactor below.

**Table 2:** SBR cycle steps for R1, R2 and R3. All reactors were operated as bottom-fed SBRs. The idle phase was used to synchronize the reactors for the next cycle.

Step	1Sedimentation[min]	2Decant 8 m^3^→4.5 m^3^[min]	3Decant and plug flow[min]	4Fill4.5m^3^→8 m^3^[min]	5Mixing[min]	6Aeration[min]	7Sludge wasting0.75–1.5 m^3^[min]		8Idle mixing(Synchronization)[min]
R1-Carb	45	45	45	45 (aerobic, DO = 0.8 mg/L)	60 (aerobic, DO = 0.8 mg/L)	-		2–5	0–300
R2-PN	45	45	45	45	30 or 60 (anaerobic)	45–300 (nitritation, DO = 1–5 mg/L)		-	0–35
R3-AMX	15	45	45	45	20 (anoxic)	30–120 (ammonium polish, DO = 0.8–1.2 mg/L)		-	0–340 (NO_x_ polish)

C-Removal (R1-Carb): The reactor contained floccular sludge. During aerated steps a DO of 0.8 mgO_2_/L was targeted although over-aeration occasionally occurred due to biofilm formation on the DO sensor. Sludge wasting (Step 7) was manually adjusted every few weeks to reach an SRT of 1.5–2.5 d (aerobic SRT = 0.5–1 d). The idle step was terminated when R2-PN entered its decant step. The reactor was equipped with a temperature sensor (Endress+Hauser, CTS1-A2GSA) and DO electrode (Endress+Hauser, Oxymax COS61). The wastewater temperature in R1-Carb ranged from 7.4 to 22.6 °C, Fig. S2 in the SI shows the online temperature measurements. The wastewater warmed up by 1–2 °C from the time it entered R1-Carb to the time it left R3-AMX.

Nitritation (R2-PN): To reach an adequate NO_2_^−^:NH_4_^+^ ratio in R2-PN for subsequent anammox treatment, a control algorithm was implemented for the aeration step. This algorithm calculated, for each batch, a target ammonium concentration (35% of the value measured at the end of the filling step). When this target concentration was reached, aeration was stopped (Table 2, R2-PN: Aeration). The idle step was terminated when R3-PN entered its decantation step. The sludge in R2-PN was floccular and SRT was not controlled. The SRT depended on the sludge lost during the decantation step and plug flow step and varied between 10–30 d (SI, Fig. S3). The resulting total suspended solids (TSS) concentration was stable around 1.5–2 gTSS/L. The reactor was equipped with the same electrodes as R1-Carb and, in addition, had an ion selective ammonium sensor (Endress+Hauser, 71109938). More information on the start-up of R2-PN and the establishment of nitritation is found in Hausherr et al. (2022).

**Fig. 1:**
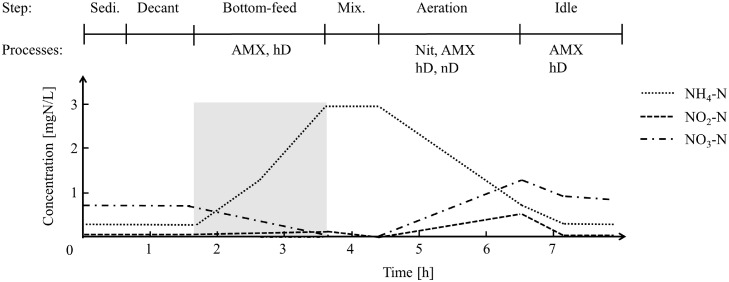
Schematic profile of dissolved nitrogen species in the anammox reactor (R3-AMX). Grey box: Concentrations are not homogenous in the reactor due to the plug flow. The drawn lines indicate the mathematically averaged concentrations. Cycle duration and absolute concentrations vary depending on the influent composition. Abbreviations: anammox (AMX), heterotrophic denitrification (hD), nitrification (Nit), nitrifier denitrification (nD).

Anammox (R3-PN): The anammox reaction occurred mainly during the reactor filling, and all nitrite was already consumed after the filling step (Table 2). Around 2–4.5 mgNH_4_-N/L remained (i.e., no more nitrite was available for anaerobic ammonium oxidation). Thus, after nitrite depletion, R3-AMX was aerated until ammonium was <1 mgNH_4_-N/L (Table 2, R3-AMX: Aeration). A schematic of the process is depicted in Fig. 1. The idle step was terminated when both R1-Carb and R2-PN had entered their idle step. Biomass grew in biofilms on carriers (FLUOPUR, Wabag) and in suspension (mostly washed in from R2-PN, 0–1.5 gTSS/L), no active sludge wasting was performed. The reactor contained 167 m^2^/m^3^ carrier surface (≈10% volume fill). R3-AMX had the same electrodes as R2-PN (DO, temperature and ammonium). R3-AMX has been running continuously since 2017; for more information on the start-up phase and anammox characterization of R3-AMX the reader is referred to Hausherr et al. (2021).

### 2.2 . Analytical methods

Wastewater samples were centrifuged (3200 g, 2 min) and filtered (0.45 µm, Macherey–Nagel) prior to analysis. Ammonium, nitrite, nitrate, and phosphate concentrations were determined using ion chromatography (Metrohm AG, 761 Compact IC and 881 Compact IC pro, Switzerland). Soluble chemical oxygen demand (sCOD), COD, total nitrogen, and total phosphorus were quantified photometrically with test kits (Hach Lange, LCK 314, 114, 338, and 348, respectively, Germany). Total suspended solids (TSS), volatile suspended solids (VSS), and the sludge volume index (SVI) were measured according to APHA et al., 2012 standard protocols.

The floc size distribution was determined by static light scattering using a Beckman Coulter (Pasadena, CA, USA) LS 13 320 particle size analyzer with a universal liquid module for the volume percentile distributions.

N_2_O was measured for 1 min every 12 min in the off-gas from the reactors with a non-dispersive infrared spectrometer (X-stream, Emerson, St. Louis MO, USA) in parts per million (ppm_v_). N_2_O is assumed to be an ideal gas, where 1 mole (44 gN_2_O/mol) occupies 22.7 L under standard conditions. The total amount of N_2_O-N [g] emitted per SBR cycle is calculated by multiplying the N_2_O concentration with the air flow rate and the length of aeration.

N_2_O emission are expressed as an emission factor (EF), which is a percentage of the total nitrogen load in the influent.

(1) $$EF_{TN,\;Influent}=\;\frac{N_{2}O\text{--}N_{emitted,D}}{TN_{Influent}*Q}\;$$

Where N_2_O-N_emitted,D_ is the cumulative N_2_O emissions per day [gN/d], TN_Influent_ is the total nitrogen concentration in the influent [gN/m^3^] and Q is the influent flow rate [m^3^/d].

### 2.3 Activity measurements

#### 2.3.1 AOB and NOB activity

Every 1–3 days two grab samples were taken during the aerated phase of R2-PN. The first sample was taken 5–20 min after aeration had started and the second sample was taken towards the end of the aeration phase. NH_4_^+^, NO_2_^−^ and NO_3_^−^ concentration were then measured to determine ammonium oxidation-, nitrite accumulation- and nitrate production-rates. The same procedure was applied for the measurement of nitrifier activity during the aerated phase of R3-AMX (Fig. 1, aeration).

#### 2.3.2 Anammox activity

During regular reactor operation, the anammox reaction occurred mainly during the bottom-feed period, in which the wastewater from the nitritation reactor flowed upward through the carriers in R3-AMX (Fig. 1, grey box). Nitrogen species could not be measured during this feeding step (due to the inhomogeneity during the plug flow). By the end of the feeding step, nitrite was usually already consumed (Fig. 1, mix.). Therefore, to measure the anammox activity, *ex-situ* batch experiments were performed.

For *ex-situ* batch experiments 750 g (wet weight) of carriers were removed from R3-AMX and resuspended in 12 L (169 m^2^/m^3^) of effluent from R3-AMX (containing < 2 mgTN/L and <30 mg soluble COD). The batch reactor was stirred until oxygen was low (DO < 0.1 mg/L). Then, 15–30 mgNH_4_-N/L as NH_4_Cl and 15 mgNO_2_-N/L as NaNO_2_ were added to the reactor. Sampling was started 15 min after NH_4_^+^ and NO_2_^−^ addition. Two to three samples were taken at 20–30 min intervals and the NH_4_^+^, NO_2_^−^, and NO_3_^−^ were analyzed, and rates determined from concentration changes.

#### 2.3.3 Inorganic nitrogen calculations

Inorganic nitrogen mass balances showed that nitrogen removal occurred during the aerated phase of R3-AMX (Fig. 1). The difference in total inorganic nitrogen concentration (ΔTIN, mgN/L) was calculated between the start and end of the aeration phase (Eq. (2)) and normalized to the consumed ammonium (Eq. (3)).

(2) $$\Delta TIN={\left(NH_{4}^{+}+NO_{2}^{-}+NO_{3}^{-}\right)}_{Initial}-{\left(NH_{4}^{+}+NO_{2}^{-}+NO_{3}^{-}\right)}_{End}\;$$

(3) $$\Delta TIN_{\%,NH4}=\frac{NH_{4,Initial}-NH_{4,End}}{\Delta TIN}*100$$

### 2.4 N_2_O experiments and isotope analysis

Six experiments were performed in R2-PN (*in situ*) in March 2020 to assess the impact of DO and organic substrate availability on N_2_O emissions.

#### 2.4.1 Experimental setup

The experiments were conducted during a regular SBR cycle of R2-PN, i.e., the reactor was filled, stirred anaerobically, and then aerated. The wastewater temperature was not controlled and ranged from 12.5 to 14.9 °C. At the beginning of the experiments, the pH was 7.5–7.6 and declined to 7.2–7.3 at the end of the aerated phase. At the beginning of each of the experiments NH_4_Cl was added to R2-PN to reach an ammonium concentration of 25 ± 2 mgNH_4_-N/L. In experiments 1–3 the blower frequency was set to 60, 50, and 40 Hz, respectively, resulting in an aeration rate of 30.5, 23.6 and 13.2 m^3^ air/h. In experiments 4–6 the blower frequency was always at 60 Hz (i.e., 30.5 m^3^ air/h). In experiment 4 and 5, 1 m^3^ effluent from the primary settler was pumped into R2-PN before (Exp. 4) or just after (Exp. 5) the anaerobic phase. In experiment 6, four times 0.25 m^3^ effluent from the primary settler was pumped into R2-PN during the aerated phase (SI Fig. S3, numbered rectangles).

#### 2.4.2 N_2_O quantification

N_2_O concentrations [ppm] were analyzed continuously in the headspace of R2-PN with a non-dispersive infrared spectrometer (X-stream, Emerson, St. Louis MO, USA). For these experiments the emitted N_2_O was normalized to the total ammonium consumed (Eq. (4)) during nitritation (since all experiments were started with the same ammonium concentration) and not to the total nitrogen in the influent of the WWTP (see, Eq. (1)).

(4) $$EF_{NH4-Consumed}=\;\frac{N_{2}O---N_{emitted}}{NH_{4,Start}*V_{R}-NH_{4,\;End}*V_{R}}$$

Where N_2_O-N_emitted_ is the total N_2_O emitted during one SBR cycle [gN], NH_4,Start_ and NH_4,End_ are the ammonium concentrations at the start and the end of the batch [gN/L] and V_R_ the reactor volume (8000 L).

#### 2.4.3 Sampling and isotope analysis

For each experiment two off-gas samples were collected in 24 liter aluminum coated gas bags (model GSB-P/44, Wohlgroth AG). One bag was filled during the first half of the aeration phase, and a second bag during the second half (SI, Fig. S4, braces). The collected gas was later analyzed by quantum cascade laser absorption spectroscopy (QCLAS) for N_2_O isotopocules (isotopically substituted molecules, e.g., ^15^N-^14^N-^16^O, ^14^N-^15^N-^16^O, and ^14^N-^14^N-^18^O); more details in (Ibraim et al., 2018). The abundance of the different isotopocules is expressed on an international scale, i.e., for ^14^N/^15^N atmospheric air N_2_ (AIR-N_2_) and for ^16^O/^18^O Vienna Standard Mean Ocean Water (VSMOW) (Mohn et al., 2016; Toyoda and Yoshida, 1999) in the conventional delta notation as follows:

(5) $$\delta \left(*\#*\right)=\;\frac{\left(R_{sample}\;-\;R_{standard}\right)}{R_{standard}}\;*1000$$

Where R_sample_ and R_standard_ are the ratio of the isotopically substitute and the most abundant (^14^N-^14^N-^16^O) species in the sample and standard, respectively.

Different N_2_O production and consumption pathways leave specific fingerprints on the O atom or the N atoms in the central (α) and terminal (β) position of the N_2_O molecule. The average ^15^N content of N_2_O is reported as δ^15^N^bulk^_N2O_, the preference for central over terminal position is termed site preference (SP) and are calculated as follows:

(6) $$\delta^{15}N_{N_{2}O}^{SP}=\;\delta^{15}N^{\alpha }-\delta^{15}N^{\beta }$$

## 3 Results

### 3.1 Effluent quality

**Fig. 2:**
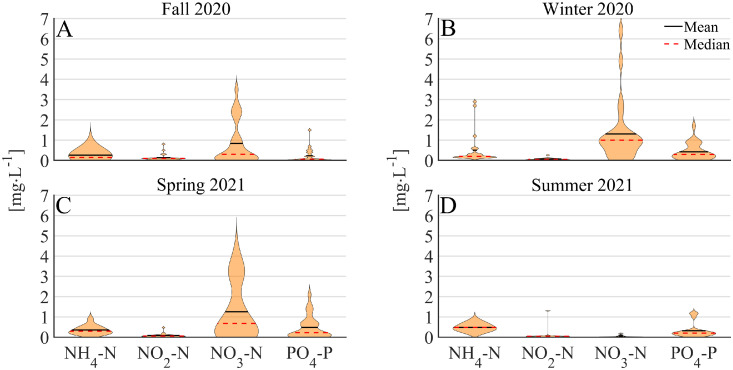
Violin plots for ammonium, nitrite, nitrate, and phosphate concentrations in the effluent from R3-AMX are shown for each season (*n* = 27, 20, 26 and 24, for A, B, C, and D, respectively).

The effluent quality, in terms of dissolved inorganic nitrogen and phosphate concentrations, of R3-AMX was assessed over a year (Sept. 2020-July 2021) and seasonal averages are depicted in Fig. 2. Ammonium concentrations in the effluent exceeded 1 mgNH_4_-N/L only during winter (3/97 measurements). Elevated nitrite concentrations of 0.5–1 mgNO_2_-N were only measured in 4/97 samples. Nitrate concentrations in the effluent were highest during winter 2020 and spring in 2021, yet, never exceeded 6.4 mgNO_3_-N/L. Anaerobic phosphate release and aerobic phosphate re-uptake, i.e., enhanced biological phosphorus removal (EBPR) took place in R2-PN (SI, Fig. S5). The effluent contained therefore generally less than 0.5 mgPO_4_-P/L. The averages and third quartile (Q_3_, i.e., 75% of values are lower than Q_3_) effluent concentrations, over the whole year, of NH_4_^+^, NO_2_^−^, NO_3_^−^ and PO_4_^3−^ are shown in Table 3.

**Table 3:** Ammonium (NH_4_^+^), nitrite (NO_2_^−^), nitrate (NO_3_^−^), and phosphate (PO_4_^3−^) concentrations in the effluent of R3-AMX are provided as average ± standard deviation (SD) and third quartile (Q_3_) over the complete study period (*n* = 97).

	NH_4_-N[mg/L]	NO_2_-N[mg/L]	NO_3_-N[mg/L]	PO_4_-N[mg/L]
Average±SD	0.4 ± 0.4	0.1 ± 0.2	0.9 ± 1	0.4 ± 0.4
Q_3_	0.5	0.1	1.1	0.5

### 3.2 Mainstream partial nitritation anammox

#### 3.2.1 Nitritation: AOB and NOB activity

**Fig. 3:**
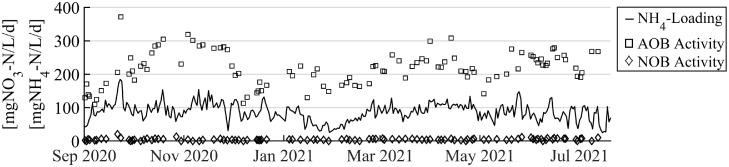
AOB activity (squares, quantified as ammonium consumption rate) and NOB activity (diamonds, quantified as nitrate production rate) during the aerated phase of the SBR cycle and ammonium loading of R2-PN are shown.

The ammonium oxidation rates in the aerobic phase of R2-PN averaged 219 ± 74 mgNH_4_-N/L/d (Fig. 3, AOB Activity). Average ammonium loading, defined as mass of ammonia per maximum reactor volume and overall cycle time, was 84 ± 43 mgNH_4_-N/L/d (Fig. 3). The AOB activity is often significantly higher than the ammonium loading rate because the aerated time is small fraction of overall SBR cycle time. Changes in NH_4_-loading were driven by the influent ammonium concentration. Especially during rain events NH_4_-loading decreased since the plug flow feeding phase and anaerobic stirring phase had fixed lengths and thus limited how much HRT could be reduced. NOB activity was successfully suppressed throughout the year, i.e., nitrite oxidation to nitrate was 40 times lower than AOB activity.

#### 3.2.2 Nitritation: ammonium conversion efficiency and the NO_2_^−^:NH_4_^+^ ratio

**Fig. 4:**
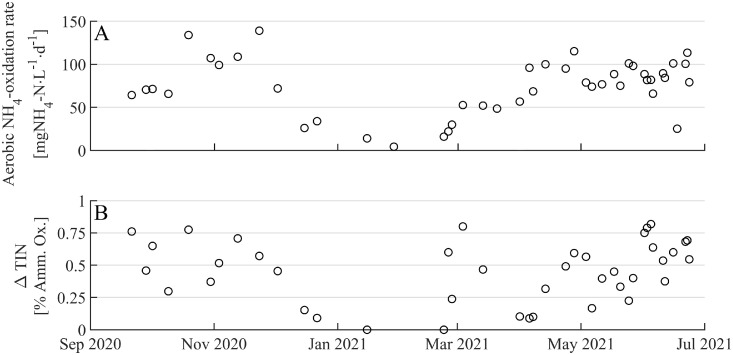
(**A**) Ammonium oxidation rates [mgNH_4_-N/L/d] in R3-AMX during the aerated phase. (**B**) The amount of total inorganic nitrogen removed during aeration (ΔTIN) (Eq. (2)) as a fraction of the ammonium oxidized (Eq. (3)).

An average ammonium to nitrite conversion efficiency (i.e., how much of the consumed ammonium is found as nitrite) of 89% was achieved in the nitritation reactor, over the full study period (SI, Fig. S6). The 11% deficit in conversion efficiency cannot solely be attributed to nitrite oxidation to nitrate by NOB (∼2.5%; Fig. 4), but is also due to denitrification (emission of N_2_ and N_2_O) and assimilative ammonium usage for cell growth.

Ammonium oxidation was stopped (i.e., aeration ceased), when 65% of the initial ammonium concentration, quantified at the onset of the aeration cycle, was consumed. Taking into account the 89% conversion efficiency of NH_4_^+^ → NO_2_^−^ an NO_2_^−^:NH_4_^+^ ratio of 1.7 is obtained in R2-PN at the end of the aeration phase (SI, Fig. S6). Limited mixing of inflow and outflow, i.e. imperfect plug flow, during the bottom feeding of the nitritation reactor and transfer to R3-AMX resulted in an estimated NO_2_^−^:NH_4_^+^ ratio in the anammox reactor of 0.8–1.4 (SI, Fig. S6).

#### 3.2.3 Anammox activity and nitrogen removal

R3-AMX had been operating, with external nitrite addition, at a N removal rate of 150–300 mgN/L/d for three years prior to this study (Hausherr et al., 2021). *Ex-situ* batch activity measurements during this study (with nitrite coming only from the nitritation reactor) showed that R3-AMX still supported high N removal rates (178 ± 43 mgN/L/d) during 2020–2021 (SI, Table S1). However, due to the imperfect plug flow the NO_2_^−^:NH_4_^+^ ratio supplied to R3-AMX was 0.8–1.4, which usually resulted in 1–3 mgNH_4_-N/L remaining after nitrite had been exhausted. This incomplete ammonium removal and the lack of nitrate accumulation (as a by-product of the anammox anabolism), clearly indicate that anammox and denitrification were co-occurring. COD for denitrification is available in R3-AMX due to the imperfect plug flow conditions in R1-Carb and R2-PN (SI, Fig. S10). The remaining ammonium was oxidized during an aeration step (4–139 mgNH_4_-N/L/d, Fig. 4**A**). Washout of nitritation-biomass from R2-PN into R3-AMX led to nitrite accumulation during the aeration step (average = 0.7 mgNO_2_-N/L, Fig. 1). Moreover, nitrogen mass balances indicated that the low DO (0.8–1.2 mgO_2_/L) allowed N removal in the deeper layers of the biofilm (SI, Table S2). The inorganic nitrogen removed during aeration (ΔTIN, Eq. (2)) was between 0–75% of the consumed ammonium (Eq. (3), Fig. 4**B**). Washout of suspended biomass from R3-AMX (SI, Fig. S7), led to the decrease of ammonium oxidation rates and TIN removal from December 2020 to February 2021 (Fig. 4).

### 3.3 N_2_O production and emission

#### 3.3.1 N_2_O emissions from R1-Carb, R2-PN and R3-AMX

**Fig. 5:**
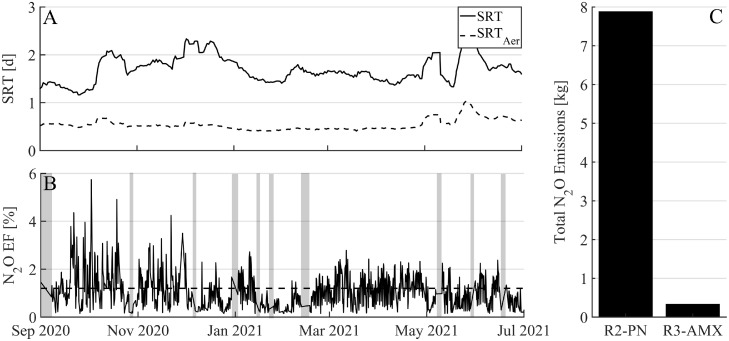
(**A**) Solids retention time (SRT) and aerobic SRT (SRT_Aer_) in R1-Carb. (**B**) N_2_O emission factor (EF) of R2-PN in [%] of total nitrogen in the influent of the pilot WWTP. Grey boxes: data could not be used or is not available due to N_2_O sensor failure, ammonium sensor drift or maintenance shutdown. Dashed-line: average EF from Sep. 2020 to Jul. 2021. (**C**) Cumulative N_2_O emissions [kg] during the aerated phase of R2-PN and R3-AMX over the complete study period.

N_2_O in the off-gas was analyzed for each reactor for 1 min every 12 min throughout the year. No N_2_O emissions were recorded in R1-Carb, where, due to the short aerobic SRT (0.5–1 d, Fig. 5**A**), ammonium oxidation did not take place, i.e., no nitrite or nitrate was available to be denitrified and possibly emitted as N_2_O. In contrast, for R2-PN high N_2_O emission factors from 0.2–6% (Eq. (1)) were measured (Fig. 5**B**). During regular reactor operation N_2_O emissions averaged 1.2% of total nitrogen in the influent (Fig. 5**B**, dashed line). In R3-AMX N_2_O was emitted during the aerated phase (ammonium polish), but cumulative emission (over the study period) from R3-AMX were only a small fraction (4%) of cumulative emissions from R2-PN (Fig. 5**C**).

#### 3.3.2 Influence of DO and organic substrate on N_2_O emissions

Six experimental conditions were tested (Table 4) to identify whether N_2_O emissions in the nitritation reactor (R2-PN) are affected by DO and organic substrate availability (Material and methods: N_2_O experiments). Decreasing the aeration rate significantly impacted N_2_O emission. More precisely, emissions increased by a factor of 3.4 (from 5 to 17.1% of the consumed ammonium, Eq. (4)), when the aeration rate was decreased by a factor of 2.3 (30.5 to 13.2 m^3^ air/h). In contrast, dosing of carbon-rich wastewater (primary settler effluent), in Exp. 4–6, did not markedly reduce N_2_O emissions. It has to be noted, that before the “Pre-Dosing” experiment, a rain-event diluted the wastewater resulting in an increased DO (5.4 mgO_2_/L), which presumably lowered N_2_O emissions (3.4%). The effect of intermittent dosing of carbon-rich wastewater was clearly indicated by sequential drops in DO (SI, Fig. S8, Exp. 6), but did not lead to an increase in complete denitrification to N_2_. In all experiments N_2_O emissions continuously rose over the course of the experiment (SI, Fig. S8) .

**Table 4:** N_2_O emissions were assessed under six different experimental conditions. N_2_O emission are provided as percentage of ammonium consumed in R2-PN. In addition the average dissolved oxygen concentrations and the nitritation efficiency are reported. Abbreviations: Dissolved oxygen (DO).

Experiment	Conditions:Aeration rate 1–3Organic substrate 4–6	Average DO[mgO_2_/L]	Nitritationefficiency[%]	N_2_O emission[%_NH4 consumed_]
1	30.5 m^3^ air/h	2.9	90	5
2	23.6 m^3^ air/h	2.1	84	6.9
3	13.2 m^3^ air/h	0.3	45	17.1
4	Pre-Dosing	5.4	94	3.4
5	Post-Dosing	2.9	92	5.3
6	Intermittent-Dosing	2.5	87	5.2

#### 3.3.3 Identification of N_2_O production pathways by isotope analysis

**Fig. 6:**
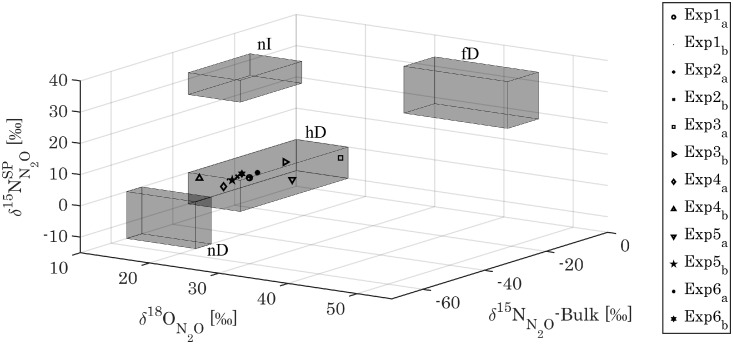
Natural isotope abundance signatures of N_2_O (*δ*^15^N^bulk^, *δ*^18^O and *δ*^15^N^SP^) of the two off-gas samples (a and b in the legend) collected for each experiment in R2-PN. Grey boxes denote expected ranges for: nitrifier denitrification (nD), heterotrophic denitrification (hD), nitrification (nI, hydroxylamine oxidation pathway), fungal denitrification (fD). Figure adapted from (Yu et al., 2020).

**Fig. 7:**
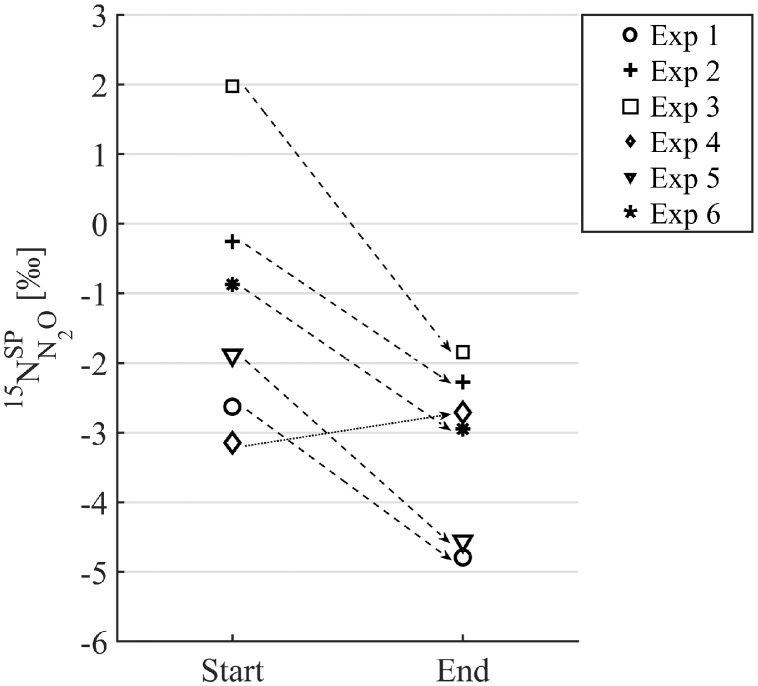
^15^N site preference (SP) of N_2_O is shown for the start and end off-gas sample during the N_2_O experiments. SP declined over the course of the experiments (dashed arrows), except for Exp. 4 (black arrow).

Natural abundance isotope signatures of N_2_O (*δ*^15^N^bulk^, *δ*^18^O, and *δ*^15^N^SP^), indicate that heterotrophic denitrification is the main process responsible for N_2_O production (Fig. 6). Low δ^15^N^SP^ reveal that the contribution of N_2_O production through the hydroxylamine oxidation pathway are minor, even in the presence of relatively high ammonium concentration (5–25 mgNH_4_-N/L) and despite high ammonium oxidation activity (i.e., compared to a mainstream flow-through N/D system). A consistent decline in δ^15^N^SP^ over the course of almost all experiments (except for experiment 4), however, might point to a minor contribution of hydroxylamine oxidation or N_2_O reduction at the onset of experiments (Fig. 7).

## 4 Discussion

The main goals of this study were to use a two-stage PNA system to clearly identify performance limitations (treatment rates and N_2_O emissions), and to attribute them to either the nitritation or the anammox stage. In contrast to other pilot-scale studies, effluent polishing was targeted.

### 4.1 Reactor characteristics and changes in N-polishing

The aerobic ammonium oxidation rates in R3-AMX decreased from December to February (Fig. 4**A**). This was linked to an observed decrease in suspended solids in R3-AMX. As a result of the decreasing biomass in suspension, ammonium polishing (ammonium < 1 mgNH_4_-N/L) took place only through AOB and NOB residing in the biofilm on the carriers and was not supported anymore through nitrifiers in suspension, i.e., the nitrification rate decreased. To increase the nitrification rate the aeration rate was increased. This ensured low ammonium concentrations in the effluent, but inhibited anoxic processes, i.e., denitrification and anammox. The enhanced aeration resulted in a 1:1 ratio of the oxidation of ammonium to nitrate without simultaneous TIN removal (Fig. 4**B**, January-February). Effluent quality was restored (i.e., low nitrate concentrations in the effluent) as suspended biomass accumulated again in R3-AMX (SI, Fig. S7). Higher rates of ammonium oxidation were possible at lower aeration rates, which re-enabled simultaneous nitrification, denitrification and anammox (Fig. 4**B**).

As reported in the supplementary material (Fig. S5), there was significant mixing of fresh influent and treated wastewater in R2-PN during the bottom-feed step (imperfect plug flow conditions). This would be detrimental to the effluent quality in R3-AMX since ammonium- and nitrite-rich influent wastewater would mix with the effluent. Interestingly, such mixing was not observed in R3-AMX (SI, Fig. S10). The carrier-bed likely dissipated wastewater currents and helped to distribute the influent evenly across the reactor footprint, enabling much better plug flow filling. A similar result was observed in a study were granular sludge led to better plug flow conditions (Yang et al., 2015). Imperfect plug flow conditions in R1-Carb are likely responsible for the EBPR in R2-PN. Phosphate release during the anaerobic phase of R2-PN was observed throughout the year except for January and February (sparse data, SI, Fig. S9). However, nitrogen removal and not phosphate removal was the focus of this study, therefore, the EBPR was not investigated thoroughly. Futures studies should assess which process is best suited for phosphate removal in PNA systems, e.g., EBPR in the nitritation stage or iron or aluminum salt addition in the carbon removal stage.

The combination of high ammonium polishing capacity in the suspended biomass and N removal capacity in the biofilm, enabled excellent effluent quality (<1 mgNH_4_-N/L and <2 mgTIN/L). However, the impact of very high influent flow rates, which need to be handled by WWTPs during rain periods, on effluent quality were not investigated in this study.

### 4.2 Nitritation rather than anammox constrained overall PNA performance

In the PNA system presented here, the wastewater is treated sequentially (C-removal, nitritation, anammox, polish). The rate-limiting step in the sequence will thus constrain the overall N-elimination performance. In this study, treatment rates were limited by the ammonium loading rates of R2-PN (84 ± 43 mgNH_4_-N/L/d, Fig. 3) rather than the N removal rates of R3-AMX (178 ± 43 mgN/L/d). Two parameters were primarily responsible for limiting nitritation. First, biomass accumulation could not be increased successfully to concentrations above 2 gTSS/L (SI, Fig. S11), at which point biomass lost in the effluent balanced biomass generation. Even though only a short settling phase (15 min) and upflow selection pressure was applied, no large particles (i.e., granules) were observed in the reactor. On the contrary, small flocs (<125 µm diameter) dominated the reactor (SI, Fig. S12), and SVI_30_ was 100–200 mL/gTSS (SI, Fig. S13), which is typical for floccular sludge. Limited biomass accumulation in R2-PN resulted in an AOB activity of 219 ± 74 mgNH_4_-N/L/d. However, in previous work, in bench-scale reactors with additional biomass retention, it was shown that rates up to 3000 mgNH_4_-N/L/d and ammonium loading rates of 400 mgN/L/d were possible (Hausherr et al., 2022). Second, long anoxic and anaerobic SBR steps prevented AOB activity throughout extended periods of the total SBR cycle. In particular, the 90% plug flow volume exchange took close to two hours (at an upflow velocity of 1 m/h) during which AOB are not active. Hence, to increase treatment rates, better biomass retention (granular sludge, an intermediary clarifier, or a membrane bio-reactor) and faster volume exchange is required. To achieve fast volume exchange, while maintaining plug flow conditions, necessitates a pipe system, which effectively distributes the wastewater across the bottom of the reactor without creating turbulence. The high-volume exchange is necessary, since it ensures high ammonium and low nitrite concentration at the start of the SBR cycle, which fosters AOB growth. In addition, lower volume exchange would result in denitrification of the produced nitrite during the anaerobic cycle phase, deteriorating the EBPR.

### 4.3 N_2_O emission patterns and scope for emission reduction

The highest N_2_O emissions were observed from the nitritation reactor, while emissions from the carbon removal stage and the anammox reactor were absent or small. N_2_O emissions were mainly modulated by nitrite concentrations, as often observed in wastewater treatment (Blum et al., 2018; Castro-Barros et al., 2016; Kuokkanen et al., 2021). This was most obvious for targeted experiments, where the nitrite concentrations rose steadily over the course of the aeration phase, and N_2_O emissions increased concomitantly (SI, Fig. S8). However, in the nitritation-system used for this study, the nitrite concentrations cannot be influenced because they are constrained by the ammonium level in the influent (each cycle 65% of ammonium needs to be oxidized to nitrite for subsequent anammox treatment). Thus, other parameters (e.g., DO and organic substrate availability), which can be influenced, were further investigated for their potential to reduce the overall N_2_O emissions.

Natural abundance N_2_O isotopes, in particular low SP values, indicate that N_2_O emissions stemmed mostly from denitrification, and not from the hydroxylamine pathway (Fig. 6) (Koba et al., 2009; Wunderlin et al., 2013). The decrease of SP over the course of most experiments, while DO steadily increased, could be related to a decreasing share of N_2_O reduction (Fig. 7), as the N_2_O molecule with the heavier ^15^N atom in the central position is slightly more resistant towards N-O-bond cleavage (Butterbach-Bahl et al., 2013). Correspondingly, in Exp. 4, where a rain event diluted the wastewater resulting in very high DO values from the beginning of the experiment (SI, Fig. S8, Exp. 4), constantly low *δ*^15^N^SP^ values were measured (Fig. 7).

Given that N_2_O production is primarily due to denitrification, increasing the aeration rate should decrease overall N_2_O production (i.e., inhibit denitrification enzymes in general). Indeed, in N_2_O experiments with high aeration rate and high DO, both N_2_O emissions and δ^15^N^SP^_N2O_ (N_2_O reduction) were minimal, indicating that reductive N_2_O production associated to partial denitrification, the prime source of N_2_O, was strongly reduced. While increasing the DO from 0.3 to 3 mgO_2_/L had a strong N_2_O mitigation effect (17.1 to 5% of ammonium oxidized in R2-PN), increasing it further to 5.4 mgO_2_/L only reduced N_2_O emissions by an additional 1.6%. To achieve high DO concentrations intensive, energy consuming aeration is required, which increases indirect CO_2_ emission from the WWTP. Yet, because N_2_O has a 298 times higher GHG potential than CO_2_, increasing electricity consumption to reduce N_2_O emissions at higher DO levels would nevertheless help in most cases to reduce the net carbon footprint of WWTPs (Liao et al., 2020). Increasing the DO is, however, not a feasible option in many nitritation system, because DO limitation is often the chosen strategy to minimize NOB activity (Isanta et al., 2015; Laureni et al., 2019). In the nitritation reactor used here, in contrast, DO limitation is not an important factor (Hausherr et al., 2022), and increased aeration could be used to reduce N_2_O emissions.

Fostering N_2_O to N_2_ reduction by increasing organic carbon availability, similar to Wan et al. (2021) did not affect N_2_O emissions nor *δ*^15^N^SP^_N2O_ (i.e. N_2_O reduction). However, if such an organic-substrate dosing strategy were conducted over longer periods, and not just once, as in the case of this study, N_2_O reduction specialists might establish in the sludge helping to reduce the net N_2_O emissions (Orschler et al., 2021; Qi et al., 2022).

With an average DO of 3–4 mgO_2_/L during regular reactor operation, an average of 1.2±1 % of total nitrogen in the influent (Table 1, 47 ± 12 mgTN/L) were emitted as N_2_O. This is close to the average N_2_O emission factor of 0.9% for N/D WWTP in Switzerland (Gruber et al., 2021). As demonstrated here, N_2_O stable isotope analyses may provide a useful analytical tool to gain a deeper understanding of biological and, possibly, abiotic N_2_O production and consumption pathways. This knowledge, in turn, will support the design and establishment of new N_2_O mitigation strategies. In addition, investigations into catalytic off-gas treatment of N_2_O are essential (Duan et al., 2021; Scherson et al., 2013). Such treatment will further reduce GHG emissions, and is even evaluated for its potential to generate energy, as for example in the coupled aerobic-anoxic nitrous decomposition operation (CANDO) process (Scherson et al., 2014).

### 4.4 Implications for PNA design

A clear consensus with regards to whether a one- or a two-stage PNA system is to be favored is still missing. The right choice of the PNA design may depend on the prioritized target (e.g., high N removal, low GHG emissions, stability) and the given framework and constraints (operator knowledge, costs or space availability), and will likely involve compromises.

To achieve high effluent quality (in particular, low effluent ammonium concentrations), one-stage PNA system currently require an additional polishing reactor, since NOB out-competition is often not stable under low ammonium conditions. In contrast, in two-stage PNA systems (operated as SBRs), ammonium polishing can be performed in the anammox stage, where NOB growth is less problematic. Indeed, as shown in this study, in the anammox stage, at low DO concentrations simultaneous TIN removal is possible, which further increases the net N-removal.

Organic shock loadings have repeatedly caused problems in one-stage PNA systems. Generally, HRAS reactors should attenuate shock loadings (Böhnke et al., 1997). But in **D**okhaven WWTP and **Sj**ölunda WWTPs, for example, organic shock loading led to PNA process disruption, even though a HRAS pretreatment was performed. Also in this study, similar to **D** and **Sj**, organic loading peaks were not completely mitigated during the C-removal stage. In the nitritation stage, they led to an increase in assimilative ammonium consumption by heterotrophic bacteria, which negatively affects the NO_2_^−^:NH_4_^+^ ratio (since ammonium assimilation lowers the nitritation conversion efficiency). Moreover, heterotrophic activity led to lower DO concentrations, which reduced AOB activity and increased N_2_O emissions (SI, Figs. S8 and S14). Thus, high organic loading resulted in reduced treatment rates for one or two SBR cycles, but very importantly, without any noticeable long-term effects.

N_2_O emissions may make up a significant fraction of the net GHG emissions of PNA-based wastewater treatment. Two-stage system may offer more flexibility for operational adjustments, such as higher aeration to reduce N_2_O emissions without affecting anammox activity. On the other hand, such adjustments might not be needed in one-stage systems, if their lower nitrite levels in general lead to lower N_2_O emission. For pilot- or full-scale mainstream PNA system little information is available regarding N_2_O emissions. Here we demonstrate that in two-stage mainstream PNA the N_2_O emission factor was similar (1%) to a one-stage system (Strass WWTP). Further reduction of N_2_O emissions is required to clearly decrease the carbon footprint of PNA systems compared to N/D systems. However, the N_2_O production and emission dynamics are still not well understood. Thus, future tasks will include the characterization of N_2_O production under various operating regimes, and the optimization of strategies to mitigate GHG emissions without compromising the N-eliminating performance of PNA systems. Once efficient off-gas treatment is viable, and routine applications is feasible, and/or more efficient strategies to reduce N_2_O emissions are discovered, two-stage PNA is a promising tool to achieve high effluent quality, and to bring WWTPs closer to energy autarky.

## 5 Conclusion

From a year-long monitoring campaign of a two-stage pilot-scale PNA system, which treated real municipal wastewater, the following conclusions are drawn:

• Suspended biomass washed out from the nitritation stage to the anammox stage allowed for PNA-based effluent polishing, resulting in very low inorganic nitrogen concentrations (average: NH_4_^+^ < 0.4 mgNH_4_-N/L and TIN <2–3 mgN/L).
• Faster plug flow filling (>1 m/h) of the nitritation stage without mixing of fresh and treated wastewater would allow for higher treatment rates than currently achieved (84 ± 43 mgNH_4_-N/L/d) and simplify reactor operation.
• N_2_O emissions averaged 1.2% of total nitrogen in the influent, higher than advanced N/D systems. Increasing the DO concentration reduced N_2_O production. But, further reduction of N_2_O emissions are required to clearly decrease the carbon footprint of PNA systems compared to N/D systems.

N_2_O isotopocule analysis showed that:

• Heterotrophic denitrification was the main process responsible for N_2_O production.
• N_2_O reduction to N_2_ was limited, and not increased significantly through the addition of organic substrate.
• Even at high ammonium concentrations (25 mgNH_4_-N/L) and high AOB activities (219±74 mgNH_4_-N/L/d) N_2_O production through the hydroxylamine pathway was negligible.

## Data availability

Data used for this study is available at the Eawag Research Data Institutional Collection (ERIC) at DOI: 10.25678/0006AH

## CRediT authorship contribution statement

**D. Hausherr:** Conceptualization, Methodology, Investigation, Data curation, Writing – original draft, Visualization. **R. Niederdorfer:** Conceptualization, Writing – review & editing. **H. Bürgmann:** Conceptualization, Writing – review & editing, Funding acquisition. **M.F. Lehmann:** Conceptualization, Writing – review & editing, Funding acquisition. **P. Magyar:** Conceptualization, Methodology, Writing – review & editing. **J. Mohn:** Conceptualization, Methodology, Writing – review & editing, Funding acquisition. **E. Morgenroth:** Conceptualization, Writing – review & editing, Supervision. **A. Joss:** Conceptualization, Writing – review & editing, Supervision, Funding acquisition.

## Declaration of Competing Interest

The authors declare that they have no known competing financial interests or personal relationships that could have appeared to influence the work reported in this paper.

## Appendix Supplementary materials

Image, application 1
