# Supplementary material for: Successful year-round mainstream partial nitritation anammox: Assessment of effluent quality, performance and N2O emissions
Source: Water Res X. 2022 Jun 16;16:100145. doi: 10.1016/j.wroa.2022.100145 (PMC9250041; doi:10.1016/j.wroa.2022.100145)
Supplement: Supplementary file 1 [file mmc1.docx]

Successful year-round mainstream partial nitritation anammox: assessment of effluent quality, performance and N_2_O emissions

D. Hausherr^1,*^, R. Niederdorfer^2^, H. Bürgmann^2^, M. F. Lehmann^3^, P. Magyar^3^, J. Mohn^4^, E. Morgenroth^1,5^, A. Joss^1^

^1^ Eawag, Swiss Federal Institute of Aquatic Science and Technology, 8600 Dübendorf, Switzerland

^2^ Eawag, Swiss Federal Institute of Aquatic Science and Technology, 6047 Kastanienbaum, Switzerland

^3^ University of Basel, Aquatic and Isotope Biogeochemistry, Department of Environmental Sciences, 4056 Basel, Switzerland

^4^ Empa, Swiss Federal Institute for Materials Science and Technology, Laboratory for Air Pollution / Environmental Technology, 8600 Dübendorf, Switzerland

^5^ ETH Zürich, Institute of Environmental Engineering, 8093 Zürich, Switzerland

*Corresponding author: Damian.hausherr@eawag.ch

**Supplementary Information**

# Materials and methods

## Bottom feeding of three coupled SBRs

A near 100% volume exchange was required for the nitritation reactor, since otherwise, much of the produced nitrite would be denitrified during the anaerobic phase of R2-PN, deteriorating the EBPR. In addition, if nitrite was still available after the “anaerobic phase” it could boost NOB growth rates. No intermediary clarifiers were available, therefore, high volume-exchange was required in all three reactors. To minimize mixing of influent with treated wastewater during the plug flow filling, the reactors were first decanted so that only ca. 3 m^3^ had to be replaced though plug flow feeding. All reactors therefore went through the following sequence: 1. Sedimentation, 2. Open effluent valve and decant, 3. Keep effluent valve open and plug flow filling, 4. Close effluent valve and fill until working volume is reached (SI, Figure S1).


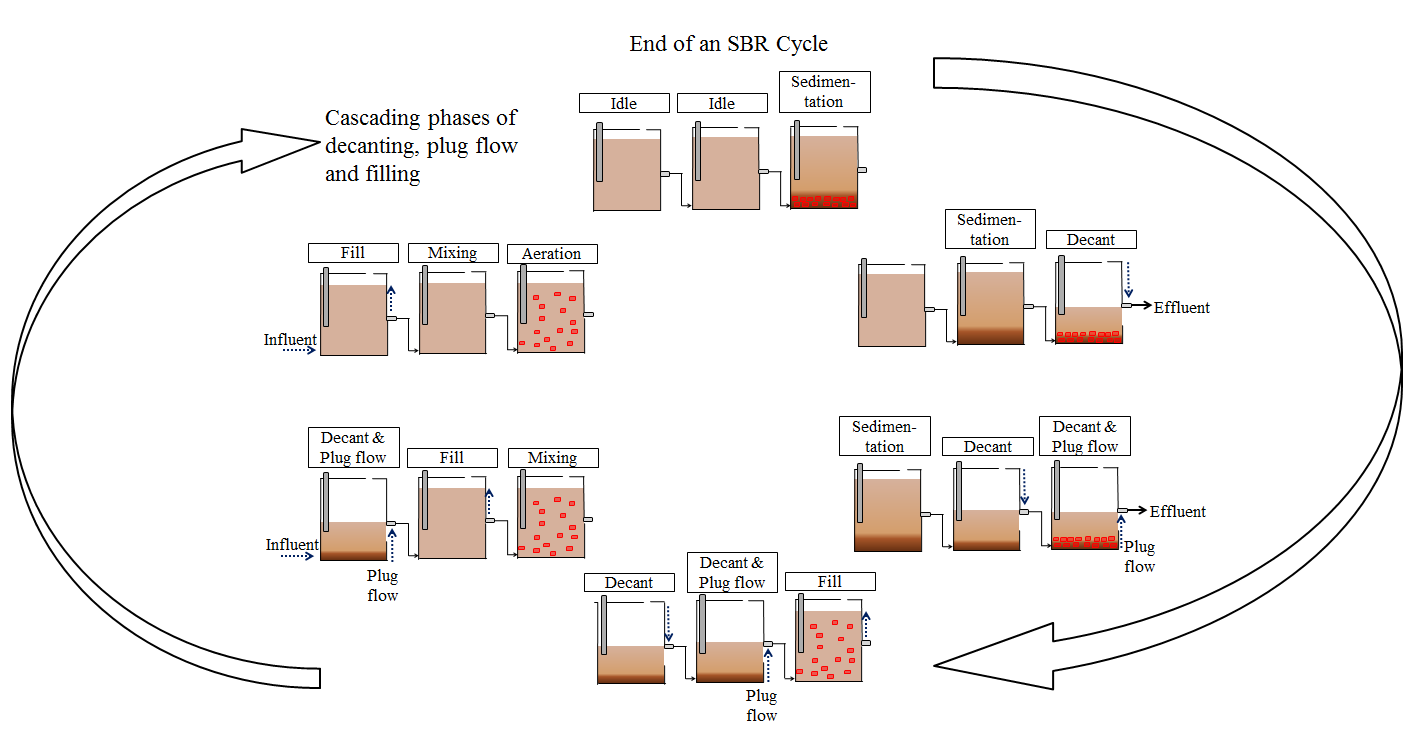


SI, Figure S1: Schematic representation of sedimentation, decantation, bottom-feed and reactor filling of the mainstream anammox treatment chain. Blue dotted arrows denote the flow of wastewater.

## Wastewater temperature profile


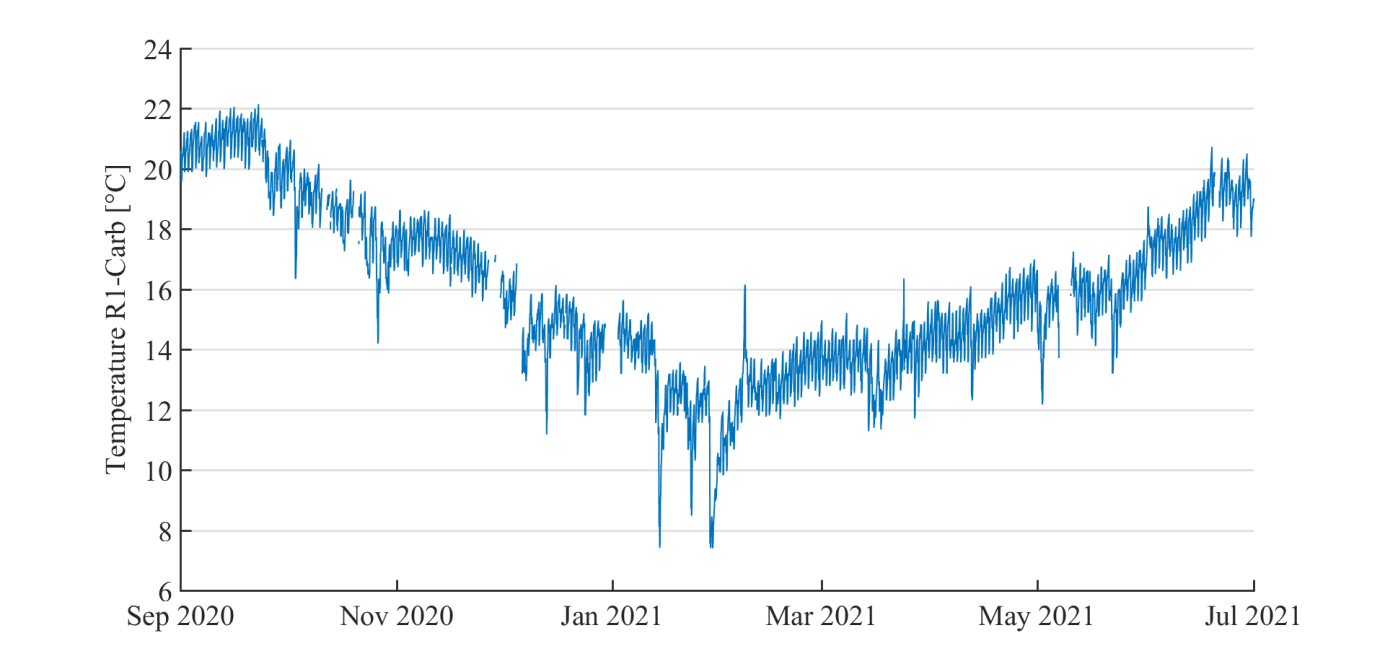


SI, Figure S2: Temperature profile of R1-Carb throughout the study period. Rains events could cause rapid temperature reduction as observed in January and February.

## SRT of R2-PN


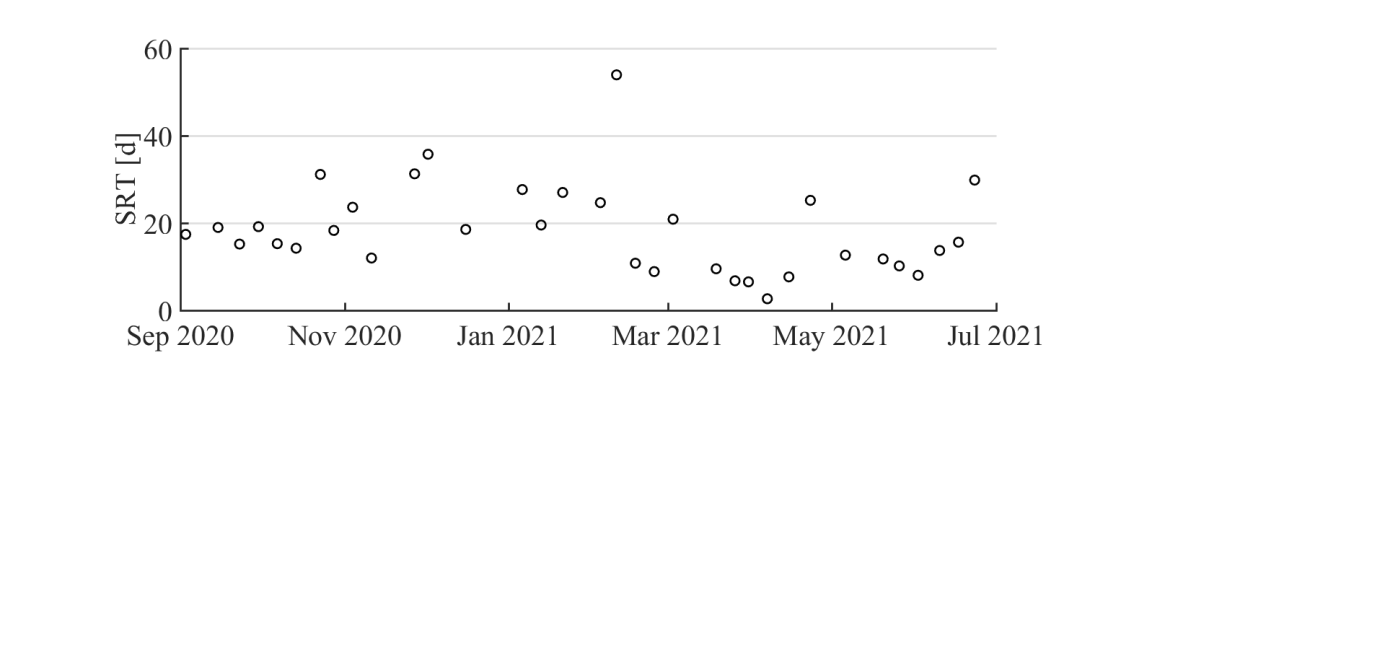


SI, Figure S3: Solids retention time (SRT) measured in R2-PN.

SRT in R2-PN was determined by the sludge lost in the effluent of R2-PN. No purposeful sludge wasting was performed.

## N2O Experiments: Dosing of organic substrate


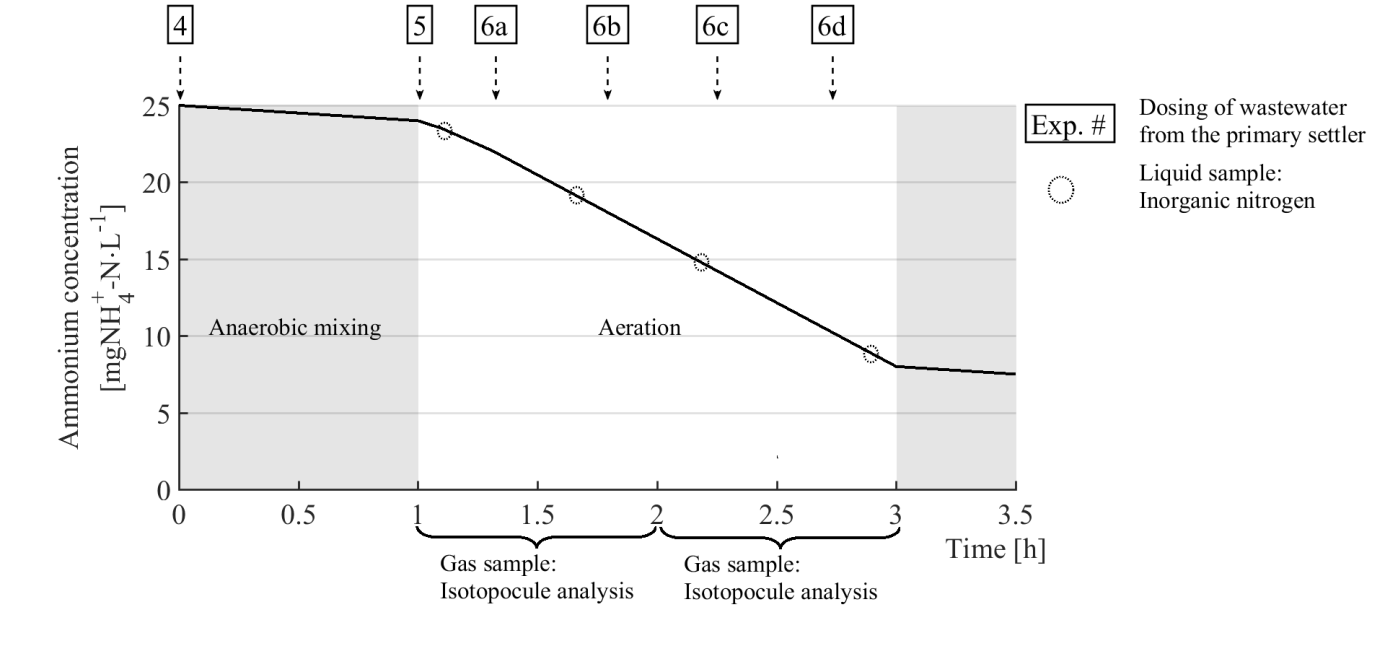


SI, Figure S4: Schematic representation of N_2_O experiments conducted during a normal SBR cycle of R2-PN. For all experiments liquid samples for inorganic nitrogen analyses (arrows) were withdrawn. Two integrated off-gas samples were collected in the first and second half of the aeration phase for N_2_O isotopocule analysis (braces). For experiments 4-6 wastewater from the primary settler was pumped into R2-PN at specific points during the SBR cycle as indicated by the numbers in rectangles. The small change in ammonium concentration due to addition of primary settler effluent is not depicted in the schematic.

# Results

## EBPR in R2-PN


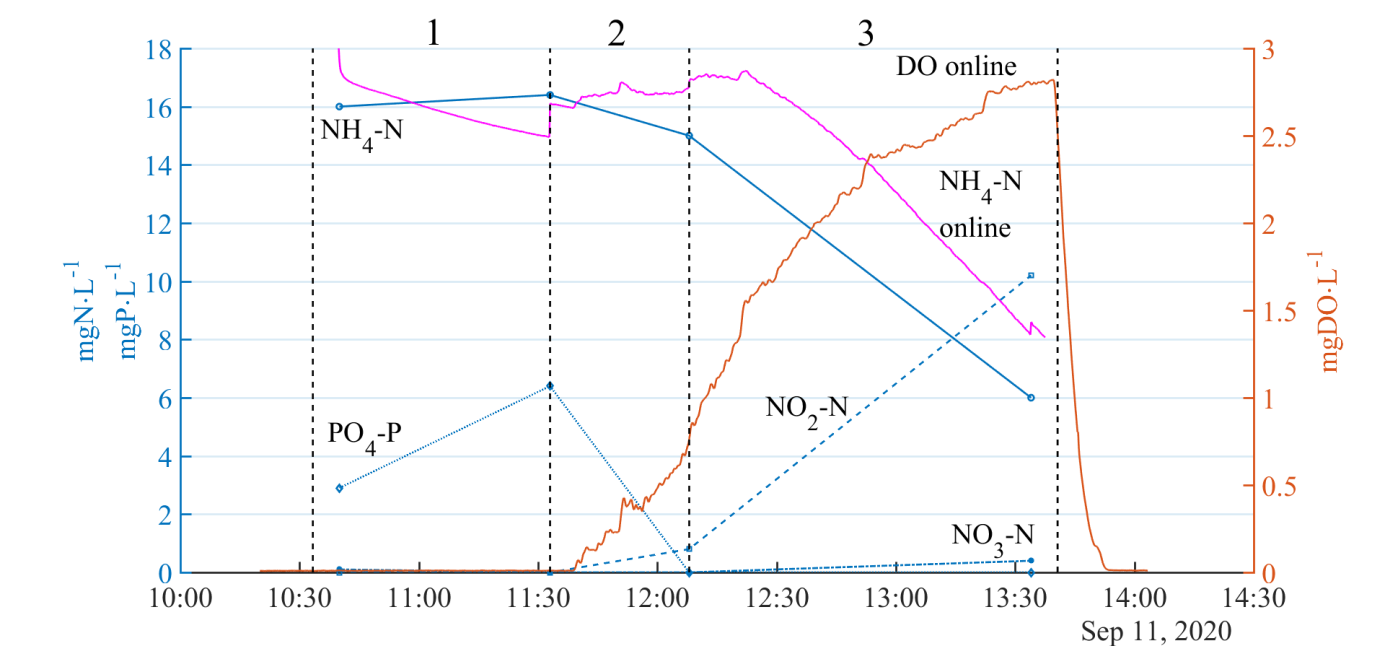


SI, Figure S5: A typical anaerobic-aerobic SBR cycle phase of R2-PN is shown. 1) During the anaerobic phase phosphate is released. 2) At the beginning of the aerated phase all phosphate is rapidly taken up by phosphate storing microorganisms. 3) After oxidation of most organic matter and re-uptake of phosphate, more oxygen is available for ammonium oxidation.

## Ammonium conversion efficiency and the NO_2_:NH_4_ ratio


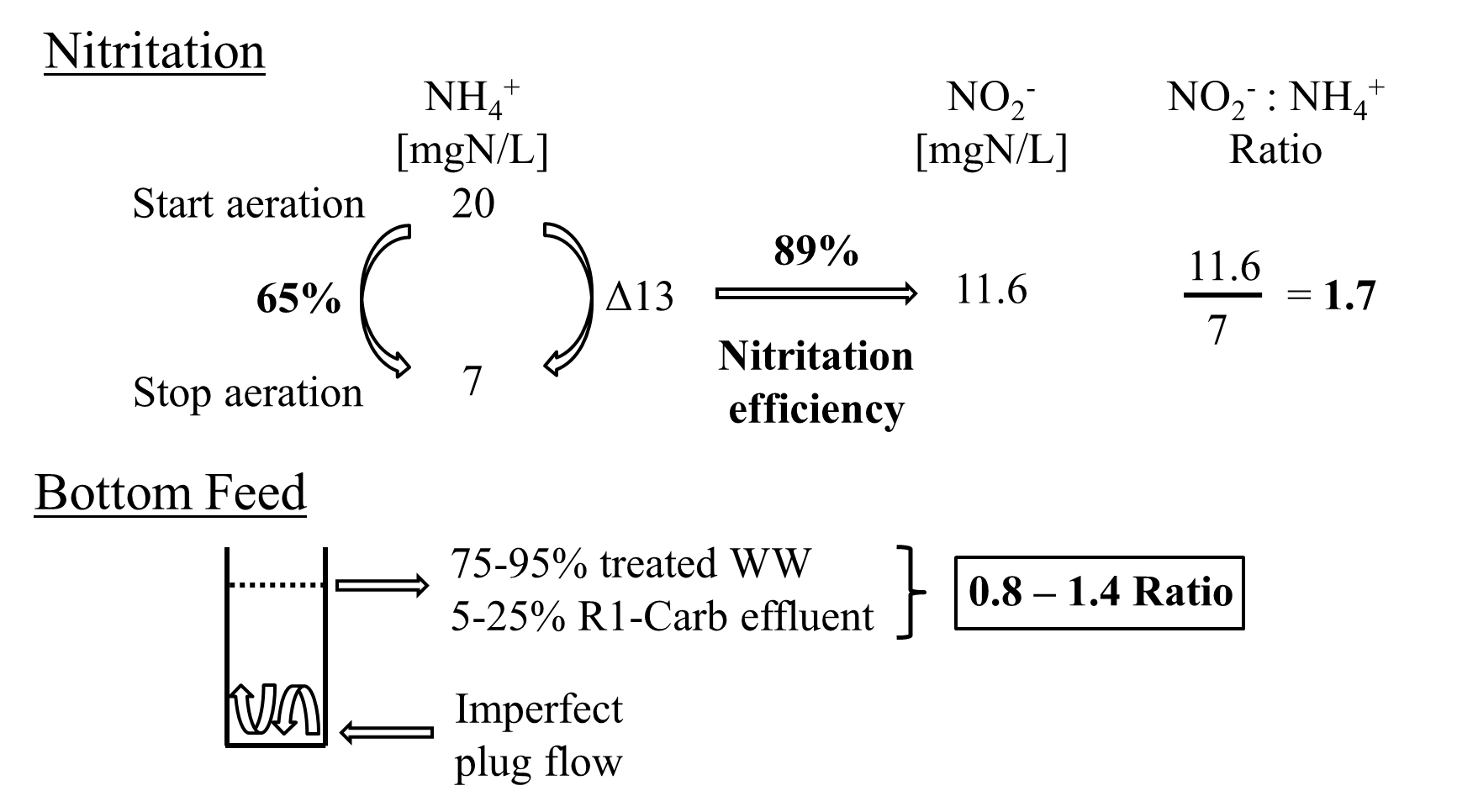


SI, Figure S6: A schematic representation of the ammonium conversion to nitrite in R2-PN to achieve a NO_2_^-^:NH_4_^+^ ratio in the effluent amenable to subsequent anammox treatment. The ratio of treated wastewater and by-pass (i.e., R1-Carb effluent) is based on estimations (assuming influent from R1-Carb containing 20 mgNH_4_-N/L and 0 mg NO_2_-N/L) since it could not be directly measured.

The imperfect plug flow had to be taken into account to achieve an NO_2_^-^:NH_4_^+^ ratio amenable to anammox. However, if ideal plug flow is achieved, aeration in R2-PN could simply be terminated after 60% of ammonium oxidation (instead of 65%), which would result directly in an NO_2_^-^:NH_4_^+^ ratio of 1.3 (assuming an 89% nitritation efficiency). Moreover, the imperfect plug-flow, e.g., the mixing of treated and fresh wastewater could significantly change between cycles (as well as the influent ammonium concentration). Thus, the final NO_2_^-^:NH_4_^+^ ratio is not well constrained which necessitates polishing capacity in R3-AMX.

## Anammox activity

Since anammox activity could not be precisly assessed during the plug flow filling of R3-AMX, *ex-situ* batch experiments were performed. Results are shown in SI, Table S1. Inorganic nitrogen concentrations were measured and the ratio of consumed NO_2_^-^:NH_4_^+^ as well as produced NO_3_^-^ per consumed NH_4_^+^ were calculated. According to the ratios, the nitrogen removal rates are likely a slight overestimation, since the pure anammox process should only consume 1.3 nitrite per ammonium and produce 0.26 nitrate per ammonium (i.e., heterotrophic denitrification slightly increased nitrite consumption and decreased the net nitrate production). However, deviations from the ideal anammox ratios have been reported in stressed anammox populations, thus it might also be an artifact of the *ex-situ* batch assays.

SI, Table S1: Data for *ex-situ* batch experiments for AMX activity.

| Date | Time | NH_4_^+^ [mgN/L] | NO_2_^-^ [mgN/L] | NO_3_^-^ [mgN/L] | NO_2_^-^:NH_4_^+^ consumed | NO_3_^-^:NH_4_^+^  produced | N-Removal [mgN/L/d] |
| --- | --- | --- | --- | --- | --- | --- | --- |
| 02.11.2020 | 13:19:00 | 37.8 | 10.3 | 7.5 |  |  |  |
| 02.11.2020 | 16:20:00 | 29.1 | 0.6 | 9 | 1.11 | 0.17 | 134 |
| 16.03.2021 | 08:30:00 | 29.2 | 16.9 | 1.4 |  |  |  |
| 16.03.2021 | 09:43:00 | 24.5 | 8.3 | 2.6 |  |  |  |
| 16.03.2021 | 11:55:00 | 18.2 | 0.1 | 3.8 | 1.53 | 0.22 | 178 |
| 17.06.2021 | 09:28:00 | 9 | 10.9 | 0.5 |  |  |  |
| 17.06.2021 | 09:55:00 | 6.9 | 7.5 | 1 |  |  |  |
| 17.06.2021 | 11:05:00 | 2.4 | 1.3 | 1.9 | 1.45 | 0.21 | 220 |

## Inorganic nitrogen mass balances in R3-AMX during aeration

SI, Table S2: Inorganic nitrogen (NH_4_^+^, NO_2_^-^, NO_3_^-^) mass balance (exemplary data) at the start and end of the aerated phase in R3-AMX.

| SBR-Cylcle | NH_4_^+^  [mgNH_4_-N/L] | NO_2_^-^  [mgNO_2_-N/L] | NO_3_^-^  [mgNO_3_-N/L] | Soluble TIN  [mgN/L] |
| --- | --- | --- | --- | --- |
| Start Aeration | 3.6 | 0.02 | 0 | 3.62 |
| End Aeration | 0.3 | 0.8 | 0.9 | 2 |
| Start Aeration | 2.9 | 0.8 | 0.1 | 3.8 |
| End Aeration | 0.6 | 0.6 | 1.3 | 2.5 |
| Start Aeration | 4.1 | 0.08 | 0.3 | 4.48 |
| End Aeration | 0.9 | 0.7 | 1.7 | 3.3 |

## TSS measurements of suspended biomass in R3-PN


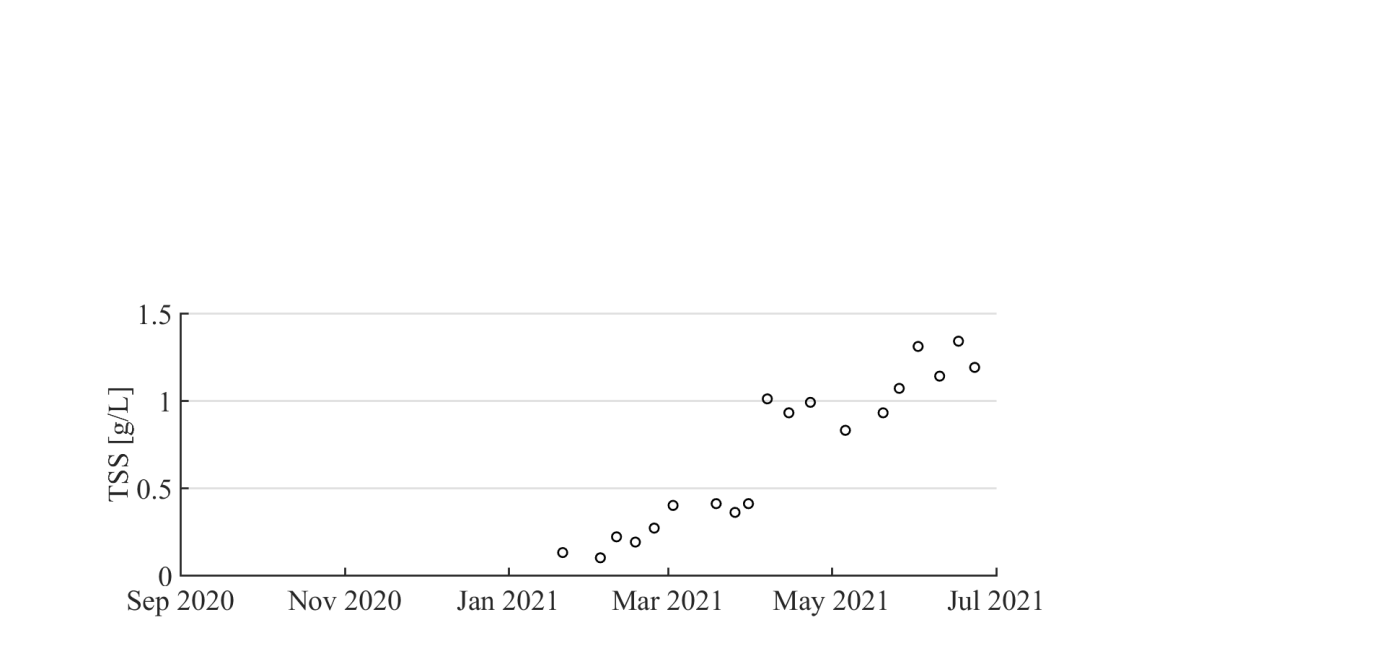


SI, Figure S7: Total suspended solids (TSS) in R3-AMX.

Initially, suspended solids were not monitored since the anammox activity resided in the carrier-associated biofilms. But after a washout-event of suspended biomass, effluent quality deteriorated, and TSS measurements were started. In April, 0.5 m^3^ suspended solids from R2-PN were pumped into R3-AMX, which increased TSS from 0.5 to 1 gTSS/L.

## N_2_O and DO profiles during N_2_O experiments in R2-PN


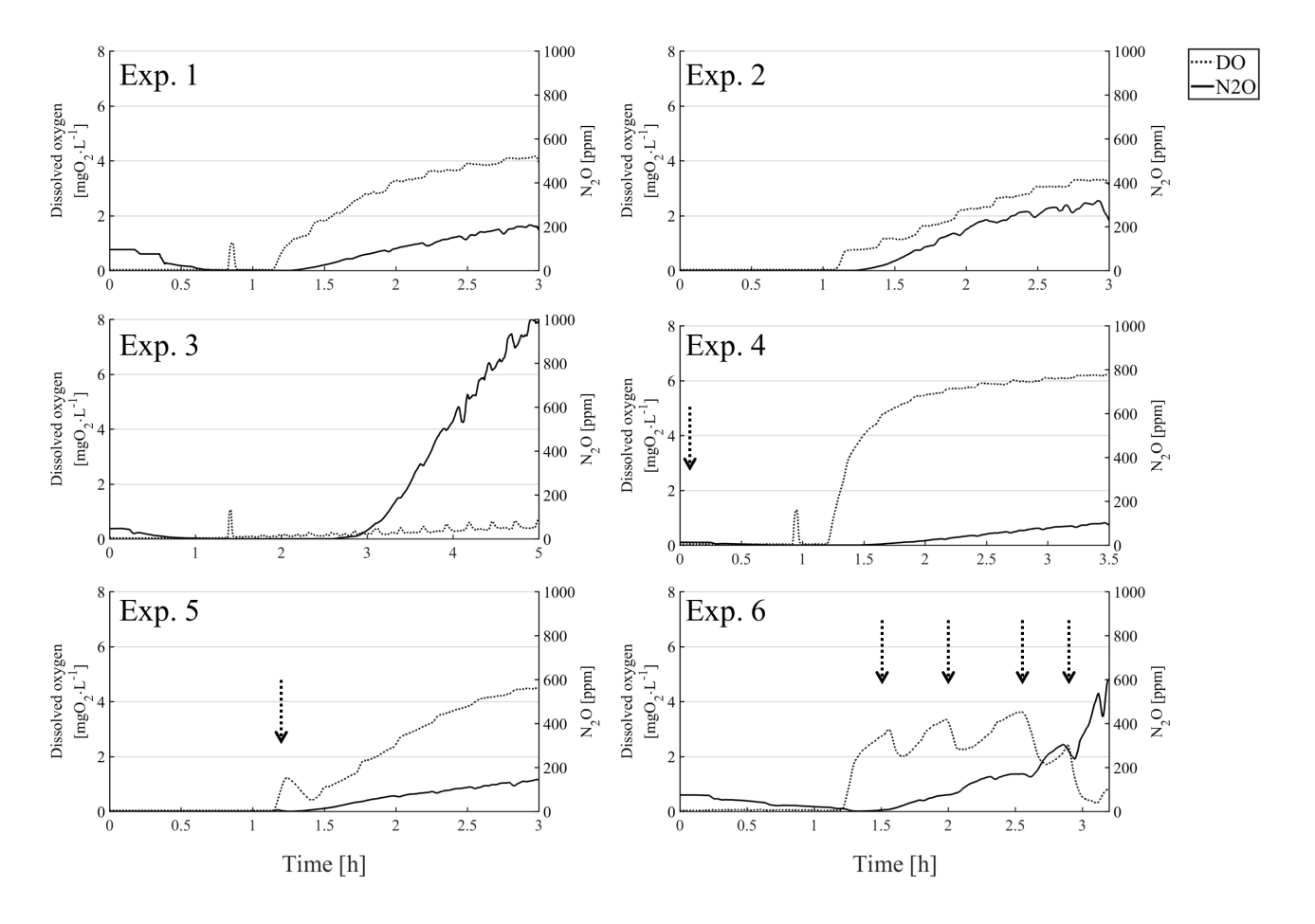


SI, Figure S8: Dissolved oxygen (DO) and N_2_O profiles during the six N_2_O experiments are shown. Dashed arrows indicate dosing of primary settler effluent. The small DO peak in Exp. 1, 3, and 4, respectively, after approximately one hour, is due to oxygen sensor cleaning.

N_2_O emission increased with time in all experiments, correlating with the increasing nitrite concentration. But, even though higher absolute nitrite concentration were present in Exp.1 compared to Exp. 3, high DO concentrations led to lower N_2_O production.

# Discussion

## EBPR in R2-PN throughout the year


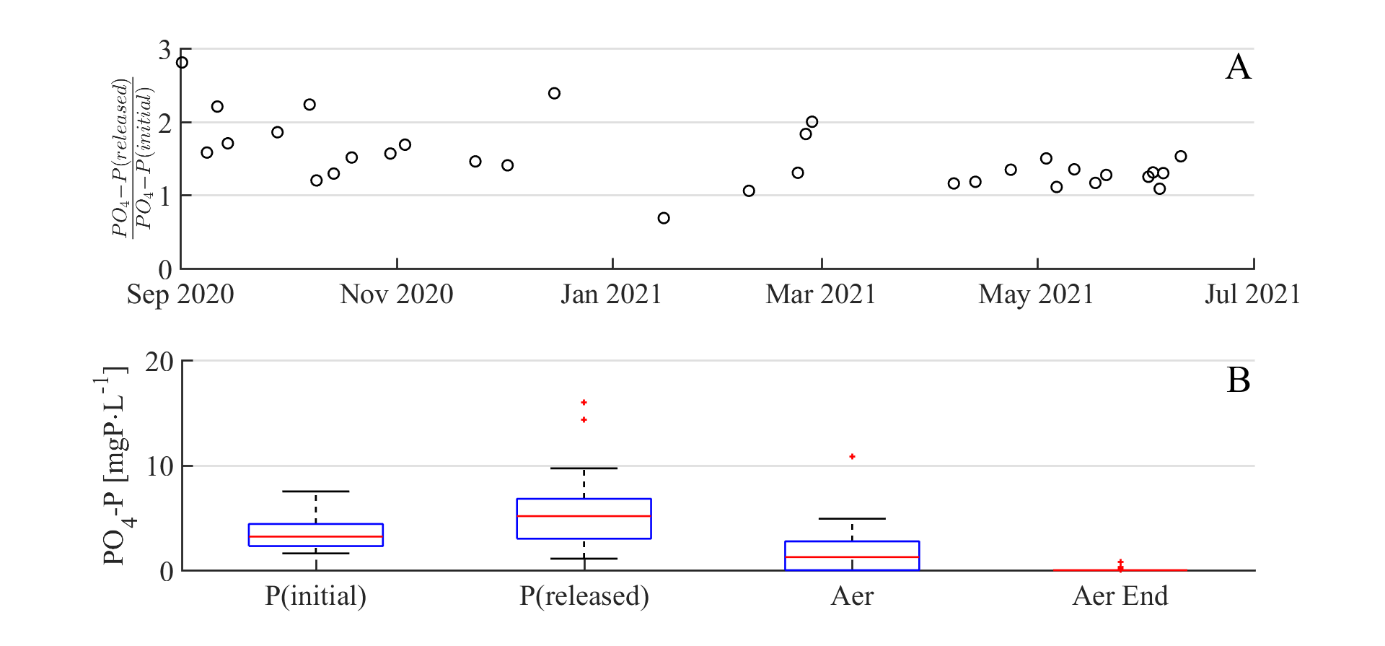


SI, Figure S9: **A**) relative phosphate release with respect to the phosphate concentration at the start of the anaerobic phase and the phosphate concentration at the end of the anaerobic phase in R2-PN. **B**) averages of phosphate concentrations: P(initial) = at the start of anaerobic phase, P(released) = end of anaerobic phase, Aer = 15-30 minutes after aeration phased started, Aer End = End of aeration phase.

## Carbon, Nitrogen and Phosphate dynamics

Due to the near complete volume exchange during each cycle, it is possible to follow a “block” of wastewater through the treatment chain.


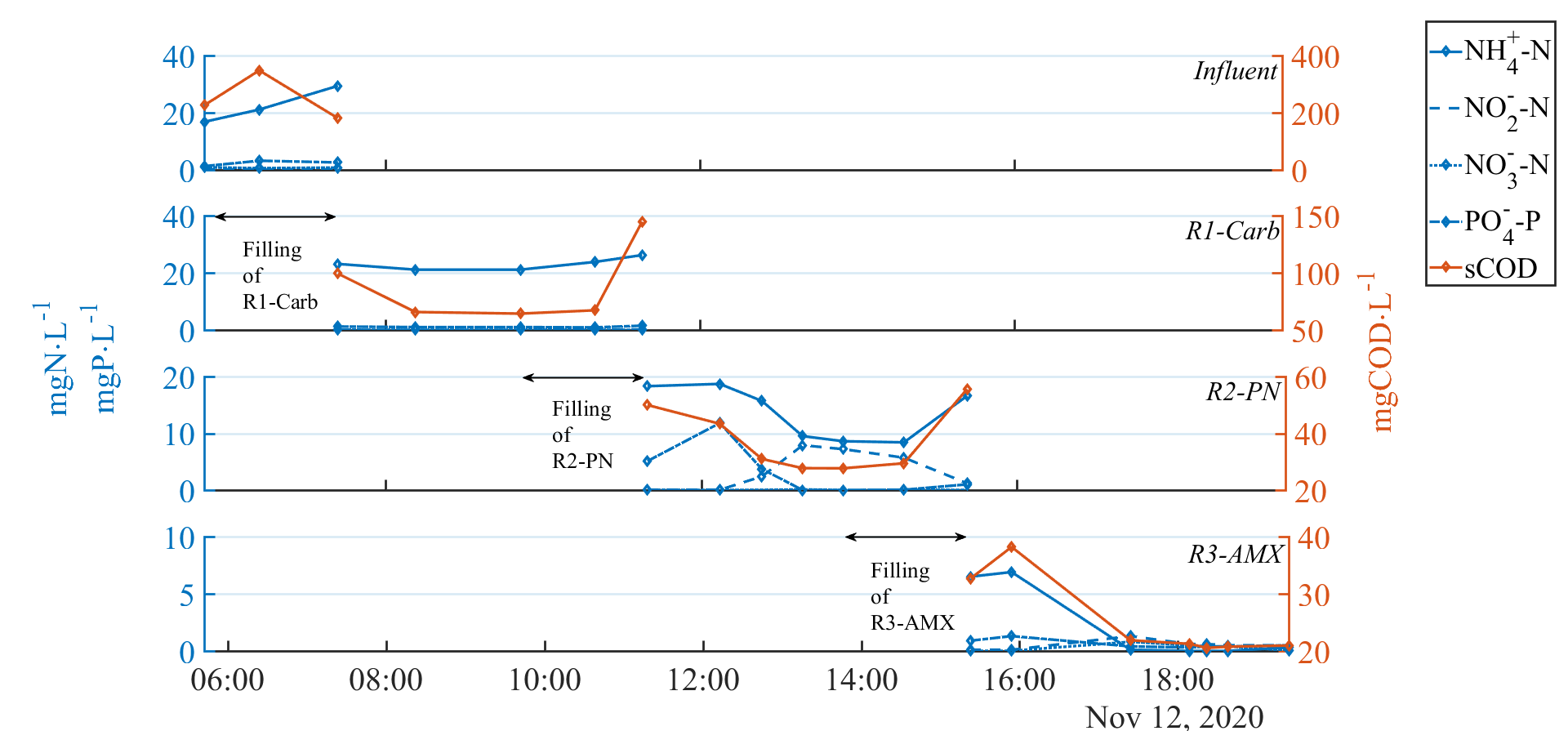


SI, Figure S10: Inorganic nitrogen, phosphate and soluble COD concentrations are shown as wastewater moves through the treatment chain (carbon removal, nitritation & EBPR, anammox & polish). The “imperfect plug flow” is clearly visible towards the end of the SBR cycle of R1-Carb and R2-PN (sudden increase in COD), whereas no such change is visible in R3-AMX.

## TSS measurements in R2-PN


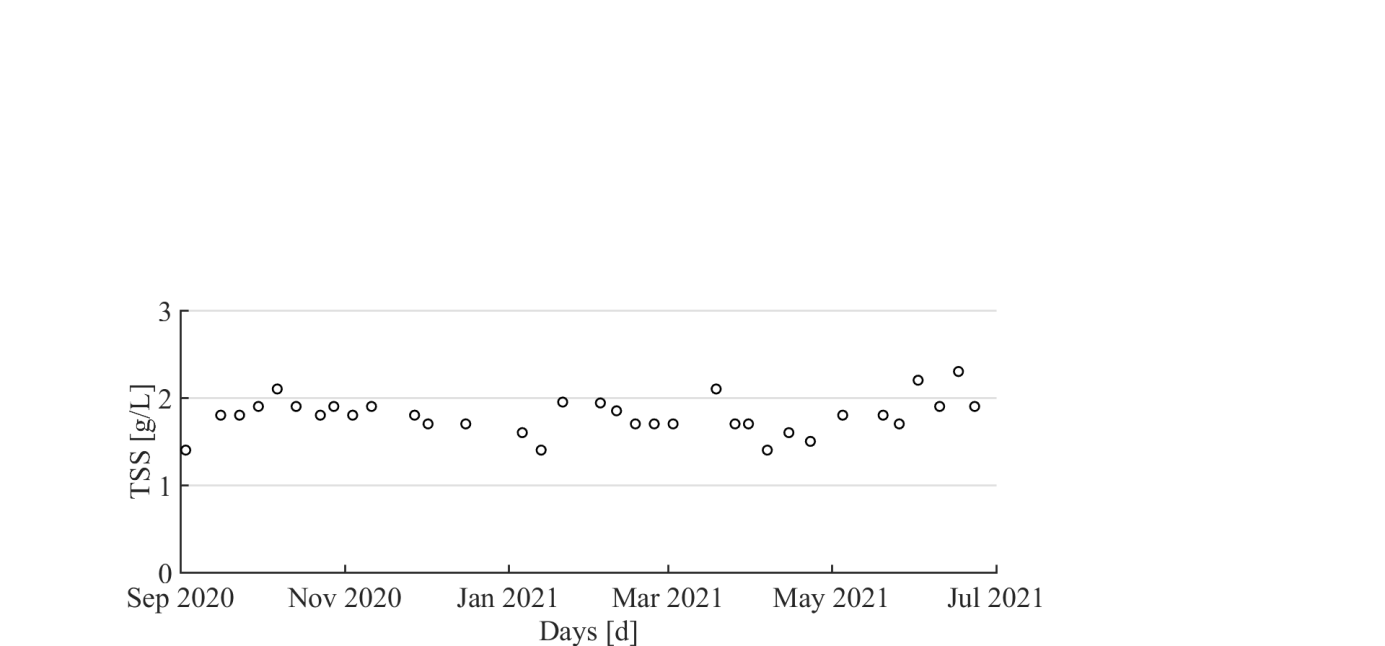


SI, Figure S11: Total suspended solids (TSS) in R2-PN.

## Size distribution and SVI measurements of R2-PN


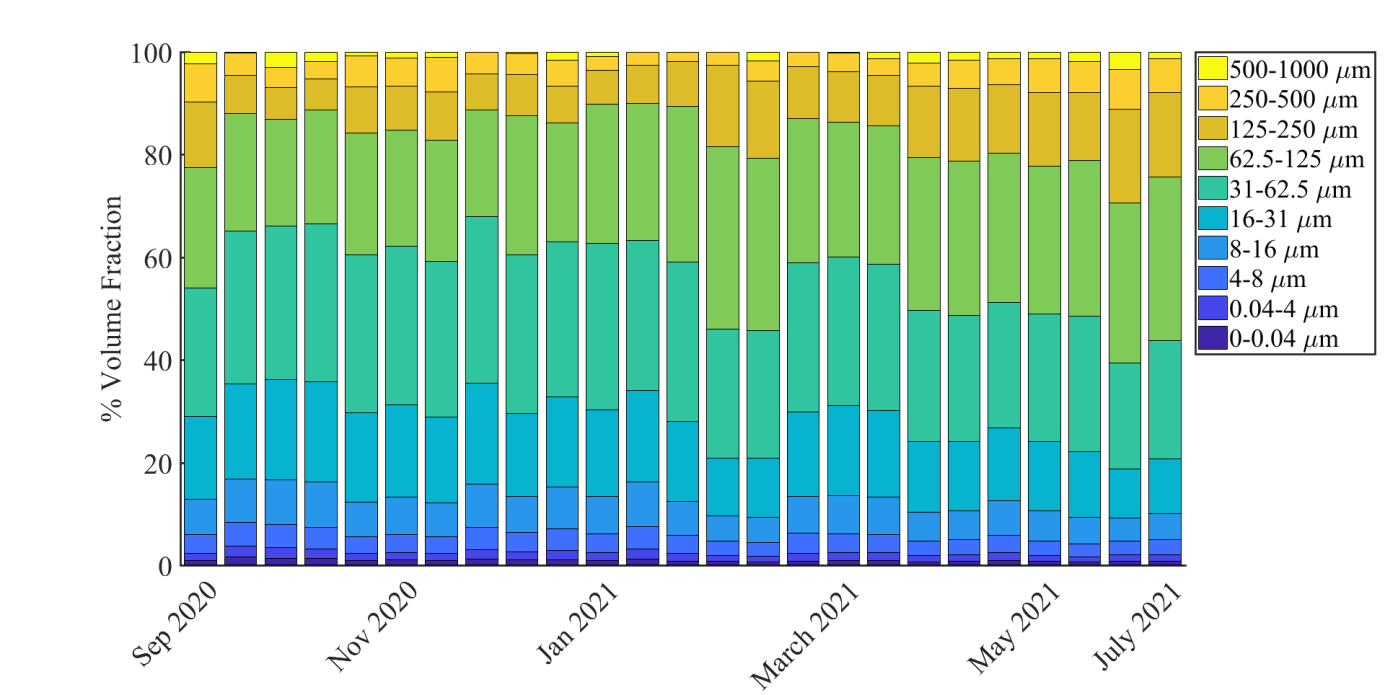


SI, Figure S12: Volume fractionation data of suspended sludge in R2-PN.

The suspended solids in R2-PN consisted largely (≈ 80%) of small particles (< 125 µm diameter), which likely settle more slowly and are being washed out frequently during decantation and bottom-feed.


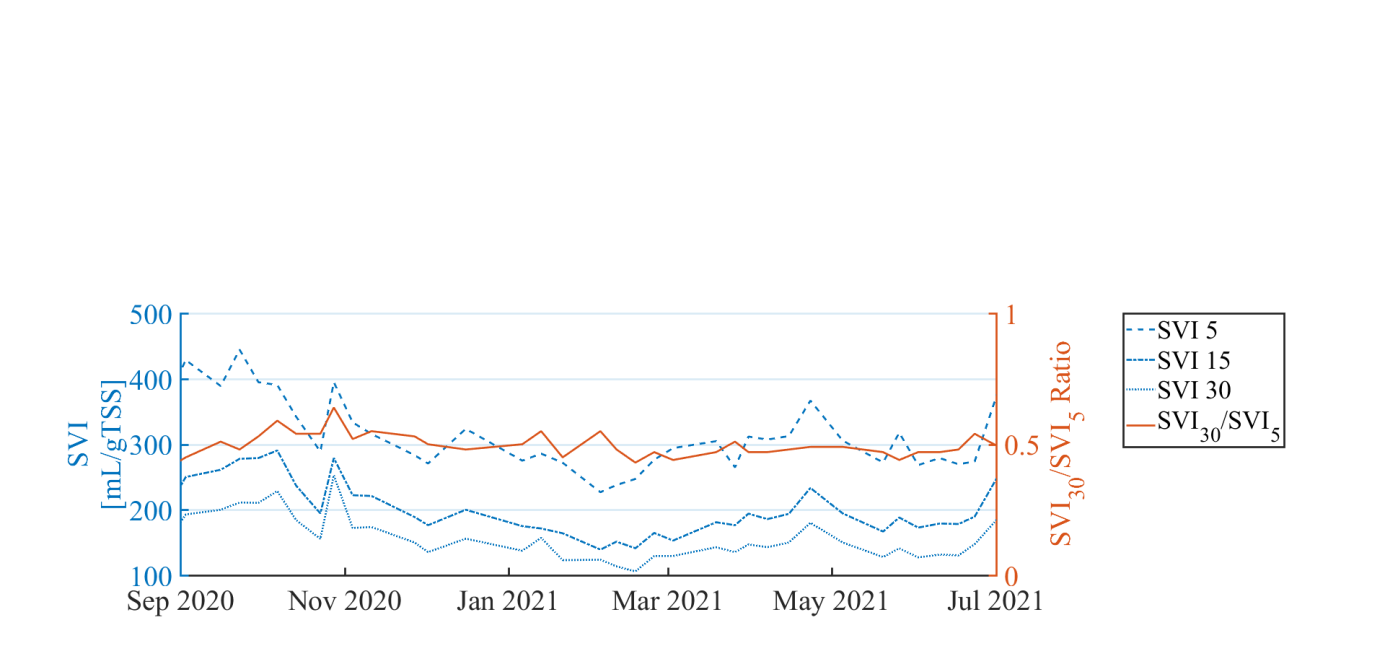


SI, Figure S13: Left y-axis: The sludge volume index (SVI) in [mL/gTSS] is shown for the 5, 15 and 30 minute mark. Right y-axis: The ratio between SVI_30_ and SVI_5_.

SVI measurements in R2-PN further indicate that the sludge was not granular, as SVI of 100-200 mL/gTSS are typical of activated sludge. Loose flocs are more easily washed out of the reactor during the bottom feeding, possibly explaining the fact that TSS could not be increased above 2 mgTSS/L (SI, Figure S11).

## Organic loading and nitritation


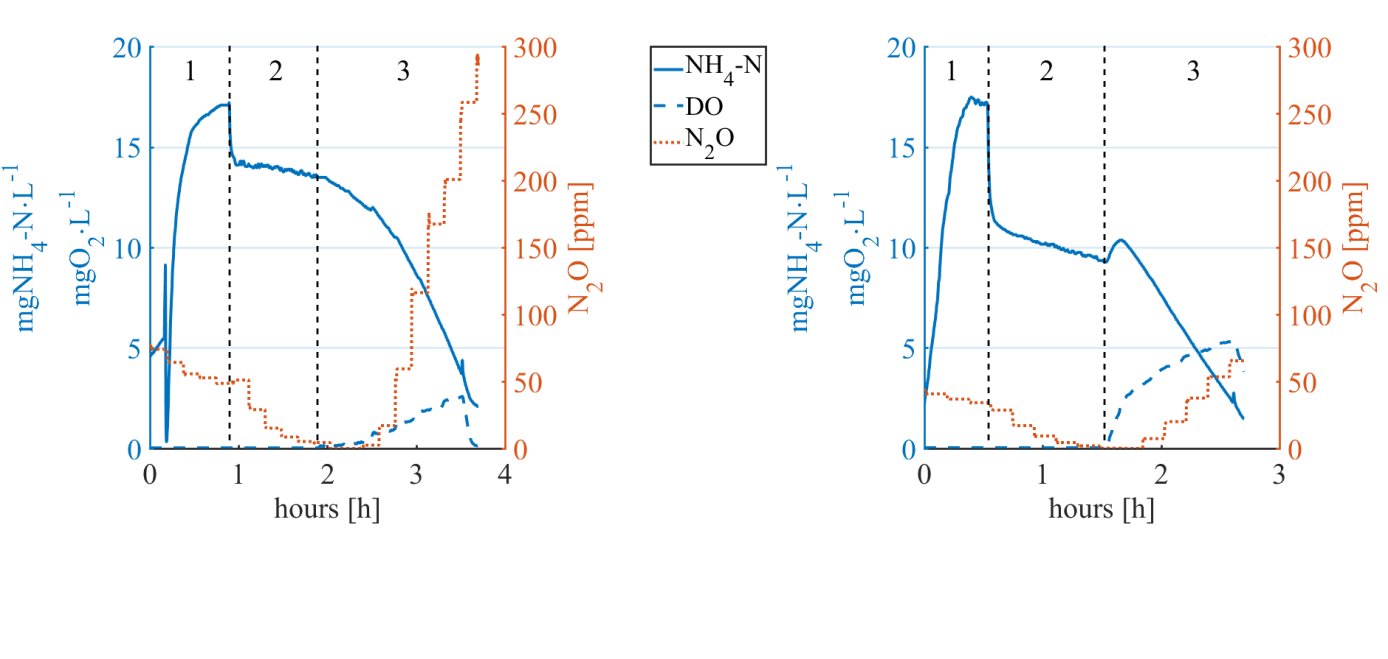


SI, Figure S14: Online measurements of dissolved oxygen (DO), ammonium concentrations and N_2_O emissions are presented for a nitritation cycle with high organic loading (left) and normal organic loading (right). 1: Reactor filling (plug flow), 2: anaerobic stirring phase, 3: aeration phase.

With high organic substrate concentrations in the influent the DO only increases to around 2 mgO_2_/L (SI, Figure S14, left), whereas 5 mgO_2_/L are reached under low organic concentrations (SI, Figure S14, right). These low DO concentrations lead to slower ammonium oxidation rates as well as increased N_2_O emissions.
